# Effects on prostate cancer cells of targeting RNA polymerase III

**DOI:** 10.1093/nar/gkz128

**Published:** 2019-03-01

**Authors:** John L Petrie, Caroline Swan, Richard M Ingram, Fiona M Frame, Anne T Collins, Hélène Dumay-Odelot, Martin Teichmann, Norman J Maitland, Robert J White

**Affiliations:** 1 Department of Biology, University of York, Heslington, York YO10 5DD, UK; 2 Université de Bordeaux, ARNA Laboratory, F-33076 Bordeaux, France INSERM, U1212 – CNRS UMR 5320, ARNA Laboratory, F-33000 Bordeaux, France

## Abstract

RNA polymerase (pol) III occurs in two forms, containing either the POLR3G subunit or the related paralogue POLR3GL. Whereas POLR3GL is ubiquitous, POLR3G is enriched in undifferentiated cells. Depletion of POLR3G selectively triggers proliferative arrest and differentiation of prostate cancer cells, responses not elicited when POLR3GL is depleted. A small molecule pol III inhibitor can cause POLR3G depletion, induce similar differentiation and suppress proliferation and viability of cancer cells. This response involves control of the fate-determining factor NANOG by small RNAs derived from Alu short interspersed nuclear elements. Tumour initiating activity in vivo can be reduced by transient exposure to the pol III inhibitor. Untransformed prostate cells appear less sensitive than cancer cells to pol III depletion or inhibition, raising the possibility of a therapeutic window.

## INTRODUCTION

Whereas pol II transcribes all protein-encoding genes, pol III synthesizes short non-coding RNAs, such as tRNA and 5S rRNA (1). Pol III is unique in having alternative paralogues of one of its subunits, POLR3G (RPC7, RPC32α) and POLR3GL (RPC7L, RPC32β), which share 49% amino acid identity (2,3). POLR3GL is ubiquitous, whereas POLR3G is enriched in stem and cancer cells (2,3). A striking decrease in expression of POLR3G when human embryonic stem cells (hESC) differentiate provoked the suggestion that it may be required for maintenance of pluripotency, perhaps through expression of specific pol III products (4). Independent studies confirmed the enrichment of POLR3G in hESC lines, as well as human induced pluripotent stem cells and very early mouse embryos, and also its down-regulation during differentiation (2,5,6). This expression pattern is consistent with evidence that the gene encoding POLR3G is directly bound and activated by NANOG and OCT4A, master regulators of stem cell pluripotency (5). Notably, hESC overexpressing POLR3G are resistant to differentiation (5,7). Conversely, RNAi of POLR3G causes hESC to lose stem cell markers and differentiate (5,7). POLR3G binds to telomerase reverse transcriptase (TERT), a protein that enhances the proliferative capacity of many stem and cancer cell types (6). The data suggest that POLR3G may suppress cell differentiation and thereby maintain proliferative capacity and phenotypic plasticity, features associated with aggressive cancers. Indeed, POLR3G can be induced by viral and cellular oncogenes and increase the growth of xenograft tumours in mice (2,6,8). These observations raise the possibility that targeting POLR3G might trigger differentiation, thereby reducing tumourigenicity. To test this, we used the PC-3 cell line, which was isolated directly from a prostate cancer metastasis (9) and displays rapid androgen-independent growth that mimics aggressive, therapy-resistant disease (10). The mainstay treatment for prostate cancer with metastatic spread is androgen deprivation, but most patients eventually develop resistance (11–13), a condition termed castrate-resistant prostate cancer (CRPC). This often involves differentiation to an androgen-independent neuroendocrine phenotype with poor prognosis (14–16). Because of the unmet clinical need for effective strategies to combat androgen-independent prostate cancer, we explored the influence of POLR3G in a model that is refractory to standard treatment. We find that depleting POLR3G can indeed promote differentiation and suppress proliferation and viability of prostate cancer cells.

## MATERIALS AND METHODS

### Cell culture and treatments

Cell lines were cultured in either RPMI medium containing 10% fetal calf serum and 5 mM glutamine (PNT2C2, BPH1 and DU145) or Ham's F12 medium containing 7% fetal calf serum and 5 mM glutamine (PC-3). Normal primary prostate epithelial cells (CC-2555) were purchased from Lonza and cultured in prostate epithelial cell growth medium bulletkit (Lonza, CC-3166). Prostate tissue was obtained with informed consent from patients (Table 1) undergoing radical prostatectomy or trans-urethral resection for prostate cancer (TURP), with approval from the local Research Ethics Committee (07/H1304/121). Epithelial cultures were prepared as previously described (17) and cultured in complete keratinocyte growth medium supplemented with 2 ng/ml leukaemia inhibitory factor, 2 ng/ml stem cell factor and 100 ng/ml cholera toxin. Patient samples were all anonymized.

**Table 1.** Clinical details

Patient number	Patient age	PSA	Operation	MRI results	Diagnosis (two samples per patient)
H627	67	7.7	Radical Prostatectomy	Adjacent Normal	
				pT2a	Gleason 7 (3+4)
H637	52	12.6	Radical Prostatectomy	Adjacent Normal	
				pT3a	Gleason 7 (3+4)
H643	78	3.4	Radical Prostatectomy	Adjacent Normal	
				pT2c	Gleason 7 (3+4)
H646	67	5.4	Radical Prostatectomy	Adjacent Normal	
				pT3a	Gleason 7 (3+4)
H455	67	0.2-0.4	Channel TURP for palliative care of advanced disease	pT3b	Gleason 9 (4+5) Metastatic bone disease.

All cells were grown at 37°C in a humidified atmosphere of 95% air and 5% CO_2_. After plating, cells were allowed to attach overnight before treatment with DMSO vehicle (‘untreated’) or the indicated concentration of ML-60218 (Cayman Chemical, Michigan, USA). RNAi was performed with Lipofectamine 3000 (Thermo Fisher) according to the manufacturer's instructions, using Silencer Select siRNAs (Thermo Fisher).

### RT-PCR

Total RNA was extracted using Trizol (Thermo Fisher), according to the manufacturer's protocol and treated with DNAse I (New England Biolabs) for 15 min at 37°C. RNA was quantified using a Nanodrop spectrophotometer and purity assessed using the 260/280 ratio. 0.5 μg RNA was reverse transcribed in a 20 μl reaction volume using random hexamers and incubation with 200 U Superscript IV (Life Technologies) at 55°C for 30 min. RT-qPCR was performed in a 20 μl reaction volume using 37.5 ng cDNA mixed with 1× Fast SYBR Green Master Mix (Applied Biosystems) and 5 μM of the primer sequences listed in Table 2 on a QuantStudio 3 Real-Time PCR System (Applied Biosystems). Denaturation step was performed at 95°C for 10 s with annealing at 60°C for 20 s and extension at 72°C for 15 s (40 cycles). Melt curve analysis was performed to ensure specificity of PCR products. qPCR analysis was carried out using Experiment Manager software provided by Thermo Fisher. The delta-delta Ct method was used for relative quantification, with normalization to the constitutively-expressed mRNA encoding acidic ribosomal phosphoprotein (ARPP) P0. NANOG-Alu-Sx primers recognize the T1 transcript described by Morales-Hernandez (18); primers for the T2 transcript from the same locus (18) gave very similar results to those shown.

**Table 2.** Primers used for real-time PCR

Transcript	Primers	Accession no./locus
pre-tRNA^Tyr^	FWD – CCTTCGATAGCTCAGCTGGTAGAGCGGAGG	chr14:21128117-21128210
	REV - CGGAATTGAACCAGCGACCTAAGGATCTCC	
Chr16.tRNA1	FWD – ATACAGCCGCAGGGCC	chr16:3200675-3200747
	REV – CGTTAACATGGCAACGAC	
Chr16.tRNA114	FWD – GGATCCTGTGGTGACCCA	chr17:66016013-66016085
	REV – CTAATCTCACGCGACCCAGATG	
ARPP P0	FWD – GCACTGGAAGTCCAACTACTTC	NM_001002.4
	REV – TGAGGTCCTCCTTGGTGAACAC	
CD55	FWD – GATGTACCTAATGCCCAGCCAGC	NM_000574.4
	REV – CATGAGAAGGAGATGGTTGCACC	
CD59	FWD – AAGGAGGGTCTGTCCTGTTCGG	NM_000611.5
	REV – GGATGAAGGCTCCAGGCTGCT	
CD63	FWD – GTGCAGTGGGACTGATTGCCGT	NM_001257389.1
	REV – GGGACTCGGTTCTTCGACATGG	
Cytokeratin 8 (CK8)	FWD – CGGAATGAATGGGGTGAG	NM_001256282.1
	REV – TGGTAGAGGCAGGAGTGGAG	
Cytokeratin 14 (CK14)	FWD – CCTCCTCCCAGTTCTCCTCT	NM_000526.5
	REV – ATCGTGCACATCCATGACC	
Cytokeratin 18 (CK18)	FWD – TGATGACACCAATATCACACGA	NM_000224.3
	REV – GGGCTTGTAGGCCTTTTACTTC	
DR2 Alu	FWD – CCTGAGGTCAGAAGTTCAAGACC	Multiple loci
	REV – GCAATGGCATGGTCTCAGCTC	
Chr2.tRNA6	FWD – ACGCACTCTAGCCTGTGGT	chr2:75124046-75124118
	REV – ACTGGTTCCCTGACCAG	
Chr2.tRNA20	FWD – CCTTTCAGCAGTTCCCATA	chr2:131094701-131094772
	REV – GCCACTTTTGTTCCCATACC	
Chr1.tRNA84	FWD – TGTCACCAGCCCTCCCT	chr1:161391883-161391954
	REV - GCAAACTTCCCTGACCG	
GRP78	FWD – GCTGAAGACAAGGGTACAGGGAAC	NM_005347.5
	REV – GGAGGGCCTGCACTTCCATAGA	
NANOG-Alu-Sx	FWD – GGGACTCGGTTCTTCGACATGG	chr12; 7783172–7783477
	REV – GGCTGATCTTGAACTCCCG	
NANOG mRNA	FWD – GATTTGTGGGCCTGAAGAAA	NM_024865.4
	REV – AAGTGGGTTGTTTGCCTTTG	
Synaptophysin (SYP)	FWD – CCAATCAGATGTAGTCTGGTCAGT	NM_003179.2
	REV – AGGCCTTCTCCTGAGCTCTT	
Neuron specific	FWD – CAATGTGGGGGATGAAGG	NM_001975.3
enolase (NSE)	REV – GTGTAGCCAGCCTTGTCGAT	
POLR3G	FWD – CACTTCGGCTGCAGAGTTTT	NM_006467.3
	REV – AGTGGGCAAATTCTGAAAG	
POLR3GL	FWD – AGAGCTACGAGGAGCCATGA	NM_032305.3
	REV – TTGTCTGAATAACGCTCCACA	
POLR3A	FWD – TCTGGAGACCTGTAGGGACAA	NM_007055.4.
	REV - CTGGCTCACCAATGCTCT	
TERT	FWD – GCCTTCAAGAGCCACGTC	NM_198253.2
	REV – CCACGAACTGTCGCATGT	
TERC	FWD – CGAGGTTCAGGCCTTTCA	NR_001566.1
	REV – CCACAGCTCAGGGAATCG	

### Northern blot

5 μg of total RNA, prepared as above, was added to an equal volume of 2× loading buffer (NEB B0363S), heated to 95°C, snap cooled on ice then run on a 10% acrylamide (19:1), 7.5 M urea, 1× TBE denaturing gel. Samples were transferred to Zeta probe GT membrane (1620196, Biorad) and UV-crosslinked using a Stratalinker on autocrosslink setting. Blots were hybridised in DIG Easy-hybe buffer (Sigma) in a hybridization oven and then sequentially probed with DIG-labelled probes designed to U2 snRNA (loading control) and tRNA_i_^Met^. Signal was developed using the DIG wash and block buffer set (Sigma) and anti-digoxigenin AP-conjugate at 1/100 000 dilution (Sigma) and CDP-star detection solution (Roche). Probe for U2 snRNA was prepared using PCR DIG probe synthesis kit (Sigma) and hybridized at 40°C. Probes for tRNA_i_^Met^ and for forward and reverse NANOG-Alu-Sx riRNA were 3′ NHS-ester DIG-labelled oligos (IDT) hybridized at 25°C. Membranes were exposed to Amersham Hyperfilm ECL, films scanned and then analysed by ImageJ software. Average fold changes of tRNA_i_^Met^ in response to ML-60218 were calculated after normalization against U2 snRNA and based on values from five independent experiments.

### Western blot

Total cellular protein was extracted using CytoBuster Protein Extraction Reagent (Merck Millipore). Protein concentrations were determined using the Pierce BCA Protein Assay Kit (Life Technologies). Proteins were fractionated by SDS-PAGE and transferred to nitrocellulose membranes, before probing with antibodies sc-28712 against POLR3G (Santa Cruz Biotechnology), HPA027288 against POLR3GL (Atlas Antibodies), 1900 against POLR3A (19) and sc-1615 against actin (Santa Cruz Biotechnology), ab53025 against NSE (Abcam), ab8049 against SYP (Abcam), ab4074 against alpha-tubulin (Abcam) and ab21685 against GRP78 (Abcam). Bands were visualised using BM Chemiluminescence Western Blotting Substrate (POD) (Roche). Recombinant POLR3G and POLR3GL were prepared as previously (2).

### Cell proliferation assays

Cells were grown on 12-well plates, treated as described and then stained with Trypan blue (Sigma-Aldrich) and counted using a Neubauer's haemocytometer.

### Immunoprecipitation

Following treatment in 15 cm dishes, protein lysates were prepared using CytoBuster Protein Extraction Reagent (Merck Millipore) and incubated overnight with preimmune serum or antiserum 1900 against POLR3A (19) pre-bound to Protein A magnetic beads (Pierce). Magnetic beads were collected and washed twice with TBST containing 0.5M NaCl and once with ultrapure water. Elution was performed by incubating magnetic beads with 50 mM glycine, pH 2 for 10 min. Eluates were neutralized with 1 M Tris, pH 8.5 and analyzed by western blot.

### Immunofluorescence

Cells were grown on round coverslips placed in 12-well plates. Following treatments, the cells were washed twice in PBS and fixed by addition of 4% paraformaldehyde for 20 min. Paraformaldehyde was removed by washing 3× with PBS, then cells were permeabilised for 10 min in 0.2% Triton-X-100. Following three washes in PBS, cells were blocked for 15 min with PBS containing 1% BSA. Cells were then incubated with antibody against Ki67 (ab15580, Abcam) for 1 h, before three subsequent washes with PBS and incubation with anti-rabbit Alexa Fluor 488 conjugated antibody (Jackson Laboratories) for 30 min in the dark. Coverslips were then washed 3× with PBS, allowed to air dry and mounted on microscopic slides with mounting medium containing DAPI (Cambridge Bioscience), for staining of nuclei. Staining was visualised using an LSM 880 confocal microscope (Zeiss) and analysed using Zen software.

### Flow cytometry

Cells were washed with PBS and centrifuged at 200 g for 5 min. Cell pellets were then resuspended in Annexin-V-FLUOS labeling solution (Roche) containing Annexin V and propidium iodide stains. Analysis was performed using a CyAn ADP flow cytometer (Beckman Coulter) and Summit software.

### Cell viability assays

Cells were grown on 96-well plates and treated as described. AlamarBlue reagent (Thermo Fisher) was then added directly to each well as 10% of the sample volume, incubated at 37°C for 30 min and fluorescence measured using a POLARstar Optima microplate reader (BMG Labtech).

### Matrigel invasion assay

Cells were plated onto Matrigel (BD Biosciences)-coated 8μm filters in 24-well plates. RPMI supplemented with 10% foetal calf serum was used as a chemoattractant. Following 48 h treatment, cells that had invaded through the Matrigel and filters were stained with DAPI and counted.

### Chromatin Immunoprecipitation (ChIP) assays

Following treatment in 15 cm dishes, PC-3 cells were cross-linked for 10 min with 1% formaldehyde. This reaction was quenched with addition of 0.125 M glycine and incubation for 5 min. Cells were then washed, pelleted and resuspended in SDS lysis buffer. Sonication was performed using a Bioruptor sonication device (Diagenode) to shear cross-linked DNA into 200–1000 bp fragments. Protein/DNA complexes were immunoprecipitated by overnight incubation at 4°C with antibodies and then collected by incubation with protein G agarose beads for 1 h at 4°C with rotation. Complexes were washed sequentially in low salt, high salt, lithium chloride and TE wash buffers for 5 min each, before the protein/DNA complexes were eluted by incubation with buffer containing 1% SDS and 0.1 M NaHCO_3_ for 30 min. Protein/DNA crosslinks were then reversed by incubation in 200 mM NaCl at 65°C for 4 h before DNA was purified with ChIP DNA Clean and Concentrator columns (Zymo Research). Purified DNA was analysed by quantitative real-time PCR using primers GGGACTCGGTTCTTCGACATGG and GGCTGATCTTGAACTCCCG for NANOG-Alu-Sx or gene desert primers CATCCCTGGACTGATTGTCA and GGTTGGCCAGGTACATGTTT, that amplify a site at 22646488 on chromosome 2 that is 46 kb from the nearest expressed sequence (20). Antibodies used were 1900 against POLR3A (19,21), sc-47701 against POLR2A (Santa Cruz Biotechnology), sc-28712 against POLR3G (Santa Cruz Biotechnology), HPA027288 against POLR3GL (Atlas Antibodies), ab3594 against H3K79me2 (Abcam) and sc-6571 against TAF_I_48 (Santa Cruz Biotechnology).

### Patient-derived xenografts

Mouse work was approved by the University of York Animal Procedures and Ethics Committee and performed under a United Kingdom Home Office License (POB5AE607). Rag2^−/−^γC^−/−^ mice were bred in the Biology Service facility, Department of Biology, University of York. Mice used for xenografts were 6–8 weeks old and housed in individually ventilated cages. Prostate tissue was obtained, with informed written consent, from a patient (H455) undergoing palliative channel TURP for advanced CRPC. The resultant patient-derived xenograft was used at the second generation in the experiments described here and has been extensively validated (22). Fresh sample was cut into 5-mm sections, mixed with 10% Matrigel at 4°C (BD Biosciences) and immediately implanted into the subcutaneous tissues of a Rag2^−/−^γC^−/−^ mouse. A tumour grew with a latency of 302 days. With serial transplantation, the mean latency is ∼70 days with a doubling time of 28 days.

To generate single cells from xenografts, tumours were minced into small cubes (3 mm^3^) and incubated in RPMI 1640 (Invitrogen, Paisley, UK) containing 5% foetal calf serum (FCS; Invitrogen) and collagenase type 1, at 200 IU/ml (Lorne Laboratories, Reading, UK) for 20 h at 37°C. Cells were washed in Dulbecco ‘A’ PBS (Oxoid Ltd, Basingstoke, UK) and disrupted further by trituration through a 21 G blunt needle (Scientific Laboratory Supplies Ltd, Hessle, UK). Cell suspensions were then incubated in 0.05% trypsin/EDTA for 30 min at 37°C, passed through 70-μm cell strainers and a Ficoll gradient (Ficoll-Paque Plus; GE Healthcare Life Sciences, Little Chalfont, UK) to further enrich for viable cells. Murine haematopoietic and mesenchymal cells were depleted by MACS (Mouse cell depletion kit (Miltenyi Biotec).

Mouse-depleted tumour cells were treated for 48 h with 20 μM ML-60218 or vehicle control. Viable cell numbers were determined using trypan blue and equivalent numbers injected subcutaneously into both flanks of 6- to 8-week-old male Rag2^−/−^γC^−/−^ mice at limiting dilutions, together with 2 × 10^5^ irradiated STO feeder cells in Matrigel. The mice were monitored until tumours reached 1.0 cm^3^. Pairwise tests and χ^2^ tests to determine tumour initiation frequencies were calculated using ELDA software (23).

## RESULTS

### POLR3G and POLR3GL have differential effects on prostate cancer cells

**Figure 1.**
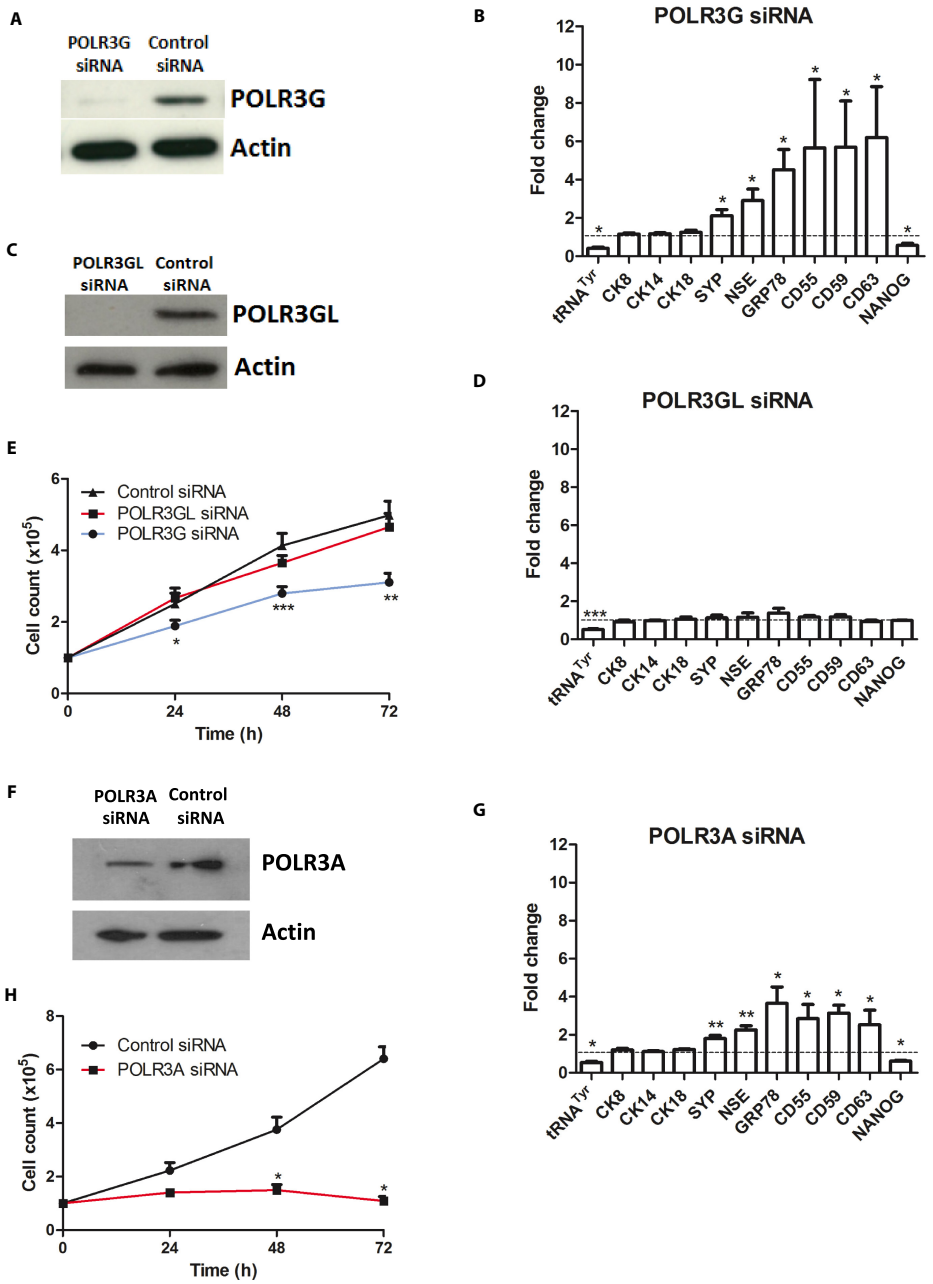
Differential response of PC-3 cells to RNAi-mediated depletion of pol III subunits. (**A**) Western blot of POLR3G and actin in PC-3 cells harvested 48 hrs after transfection with control or POLR3G-targeting siRNAs. (**B**) Mean relative changes in normalized expression of unprocessed pre-tRNA^Tyr^ and mRNAs encoding CK8, CK14, CK18, SYP, NSE, GRP78, CD55, CD59, CD63 and NANOG in PC-3 cells harvested 48 hrs after transfection with control or POLR3G-targeting siRNAs, as determined by RT-qPCR in 3–4 independent experiments. (**C**) Western blot of POLR3GL and actin in PC-3 cells harvested 48 h after transfection with control or POLR3GL-targeting siRNAs. (**D**) Mean relative changes in normalized expression of unprocessed pre-tRNA^Tyr^ and mRNAs encoding CK8, CK14, CK18, SYP, NSE, GRP78, CD55, CD59, CD63 and NANOG in PC-3 cells harvested 48 hrs after transfection with control or POLR3GL-targeting siRNAs, as determined by RT-qPCR in 3–4 independent experiments. (**E**) Mean PC-3 cell numbers over 3 days following transfection with control siRNA or siRNAs targeting POLR3G or POLR3GL, as indicated, in 5 independent experiments. (**F**) Western blot of POLR3A and actin in PC-3 cells harvested 48 h after transfection with control or POLR3A-targeting siRNAs. (**G**) Mean relative changes in normalized expression of unprocessed pre-tRNA^Tyr^ and mRNAs encoding CK8, CK14, CK18, SYP, NSE, GRP78, CD55, CD59, CD63 and NANOG in PC-3 cells harvested 48 hrs after transfection with control or POLR3A-targeting siRNAs, as determined by RT-qPCR in 3–4 independent experiments. (**H**) Mean PC-3 cell numbers over 3 days following transfection with control or POLR3A-targeting siRNAs, as indicated, in three independent experiments. * indicates *P* < 0.05 relative to control siRNA by *t*-test; ** indicates *P* < 0.01; *** indicates *P* < 0.005. Error bars represent S.E.M.

Prostate epithelial cells are classified into luminal, basal and neuroendocrine (NE) categories, each defined by expression of canonical marker genes that are transcribed by pol II. Depletion of POLR3G (Figure 1A) had minimal effect on expression of the basal cell marker CK14, or the luminal cell markers CK8 and CK18, but induced the NE markers SYP and NSE (Figure 1B). Induction of CD55, CD59 and CD63, which are detected in granules secreted by prostate (24–27), is consistent with the abundance of neurosecretory granules that characterise NE cells (15). In addition, we observed induction of GRP78, which is associated with CRPC (28). In contrast, a significant decrease was detected in expression of NANOG, a key regulator of pluripotency, stem cell self-renewal and proliferation (29–33). These data suggest that POLR3G levels can influence differentiation of prostate cancer cells, as reported previously in hESC. The response is observed at a level of POLR3G depletion that reduces expression of the primary pre-tRNA^Tyr^ transcript, a canonical pol III product, by ∼58% on average (Figure 1B).

The studies of POLR3G using hESC systems did not investigate other pol III subunits. We tested how PC-3 cells respond to depletion of POLR3GL, the paralogue of POLR3G. When POLR3GL was depleted by RNAi, pre-tRNA^Tyr^ was reduced by ∼48% on average (Figure 1C and D). Thus, both POLR3G and POLR3GL contribute in PC-3 cells to the synthesis of tRNA, an essential pol III product. This is consistent with evidence that most pol III-transcribed genes can recruit either of these subunits (3). In contrast to the similar tRNA response, depletion of POLR3GL did not suppress expression of NANOG or induce the differentiation markers that are activated when POLR3G is depleted (Figure 1D). Co-immunoprecipitation confirmed that both paralogues associate with the core pol III subunit POLR3A in PC-3 cells (Supplementary Figure S1), consistent with the comparable effects on pre-tRNA when either is depleted.

Cells generally stop dividing when induced to differentiate. Indeed, PC-3 cell proliferation was significantly reduced by RNAi of POLR3G, but not of POLR3GL (Figure 1E). Thus, selective depletion of POLR3G induces a switch from proliferation to differentiation, an effect not observed when the paralogous subunit POLR3GL is depleted to a similar extent. As synthesis of tRNA^Tyr^ is comparably reduced whether POLR3G or POLR3GL is depleted, the data suggest that the trigger to differentiate is more specific than a general decrease in total pol III output.

**Figure 2.**
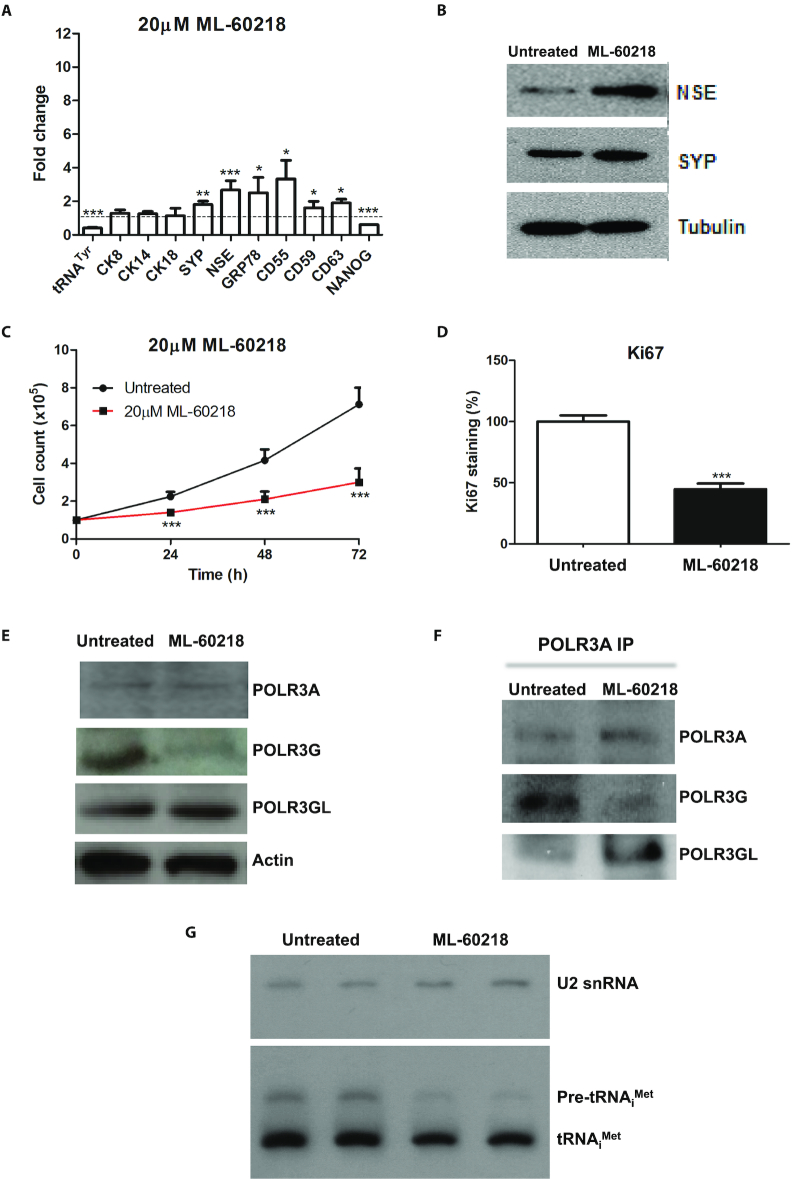
Response of PC-3 cells to pol III inhibitor ML-60218. (**A**) Mean relative changes in normalized expression of unprocessed pre-tRNA^Tyr^ and mRNAs encoding CK8, CK14, CK18, SYP, NSE, GRP78, CD55, CD59, CD63 and NANOG in PC-3 cells harvested 48 hrs after treatment with or without 20 μM ML-60218, as determined by RT-qPCR in 3–6 independent experiments. (**B**) Western blot of NSE, SYP and tubulin in PC-3 cells harvested 48 h after treatment with or without 20 μM ML-60218. (**C**) Means of PC-3 cell numbers over 3 days with or without 20 μM ML-60218, in six independent experiments. (**D**) Means of the percentage of PC-3 cells staining positively for Ki67 proliferation marker 2 days after treatment with or without 20 μM ML-60218, in three independent experiments. (**E**) Western blot of POLR3A, POLR3G, POLR3GL and actin in PC-3 cells harvested 48 h after treatment with or without 20 μM ML-60218. (**F**) Antiserum to POLR3A was used to immunoprecipitate pol III from PC-3 cells cultured for 48hrs without or with 20 μM ML-60218; panels show western blots of immunoprecipiated protein probed with antibodies to POLR3A, POLR3G and POLR3GL, as indicated. (**G**) Representative northern blot of U2 snRNA (pol II-transcribed control), unprocessed pre-tRNA_i_^Met^ and mature tRNA_i_^Met^ in PC-3 cells harvested 48 hrs after duplicate treatment with or without 20 μM ML-60218. * indicates *P* < 0.05 relative to control by *t*-test; ** indicates *P* < 0.01; *** indicates *P* < 0.005. Error bars represent S.E.M.

Although POLR3G is known to form part of the pol III enzyme complex, the possibility exists that it has additional independent functions, as yet to be discovered. Precedent is provided by aminoacyl-tRNA synthetases, many of which perform non-canonical functions unrelated to their catalytic activities (34). If POLR3G influences the balance between differentiation and proliferation as part of pol III, then it is likely to require POLR3A, the largest pol III subunit that provides much of the catalytic site (35). Consistent with this, RNAi-mediated depletion of POLR3A can also induce differentiation markers and suppress proliferation of PC-3 cells (Figure 1F–H). This suggests that the effect of POLR3G on cell fate reflects its function as part of pol III. Support for this conclusion is provided by use of ML-60218, a small molecule inhibitor with high pol III specificity (36,37). At a concentration (20μM) that decreases pre-tRNA^Tyr^ by ∼59%, similar to the siRNAs targeting pol III subunits, ML-60218 stimulates expression of SYP and NSE mRNAs, indicating that the NE differentiation programme is triggered (Figure 2A). Induction was confirmed at the protein level (Figure 2B). Dendritic processes that are characteristic of NE cells were apparent after treatment with 20μM ML-60218 (Supplementary Figure S2). Proliferation was also suppressed, as established by cell counts and staining for Ki67 (Figure 2C and D). Thus, a pol III-selective transcriptional inhibitor can mimic the effects of pol III depletion in inducing differentiation and decreasing proliferation of PC-3 cells.

Expression of telomerase provides a means to avoid the erosion of telomeres that limits replication of normal somatic cells. Both the RNA (TERC) and protein (TERT) components of telomerase decrease significantly in response to ML-60218 (Supplementary Figure S3). This is consistent with its negative effect on proliferative activity and positive influence on differentiation.

As POLR3G levels decrease when embryonic cells differentiate (2,4,5), we investigated if this is also true for PC-3 cells treated with ML-60218. Indeed, POLR3G levels are substantially lower after 48 h exposure to 20 μM ML-60218 (Figure 2E). This response is specific, as POLR3A and POLR3GL show little or no change. Although there is minimal change in POLR3GL abundance under these conditions, co-immunoprecipitation suggests that stable association of POLR3GL with the pol III core increases (Figure 2F), perhaps providing some compensation for loss of its paralogue.

The data above support a model in which cell fate decisions are influenced by pol III activity according to its subunit composition: pol III that contains POLR3G suppresses differentiation and promotes proliferation, but pol III does not have this effect when it incorporates POLR3GL instead of POLR3G. If both forms are affected by RNAi of the shared catalytic subunit POLR3A or treatment with ML-60218, loss of POLR3G-dependent function triggers the switch in cell fate that occurs when POLR3G itself is depleted; this may reflect a dominance of POLR3G relative to POLR3GL in PC-3 cells.

The most abundant pol III product is tRNA. RT-PCR detects immature pre-tRNA, but not the processed transcripts that are highly methylated and folded (38–40). Although unprocessed pre-tRNA_i_^Met^ levels decrease by 44%, mature tRNA_i_^Met^ falls by only 12% after exposure for 48 h to 20 μM ML-60218 (Figure 2G). Thus, steady-state levels of mature tRNA can be relatively stable despite significant reductions in rates of production. This is consistent with previous evidence that tRNA levels can be maintained in differentiated cells despite decreased synthesis (41). These data show that proliferative arrest and differentiation of PC-3 cells can be induced by changes in pol III activity that have only minor impact on the steady-state levels of its principle products.

### DR2 Alu transcripts are differentially regulated by POLR3G and POLR3GL

**Figure 3.**
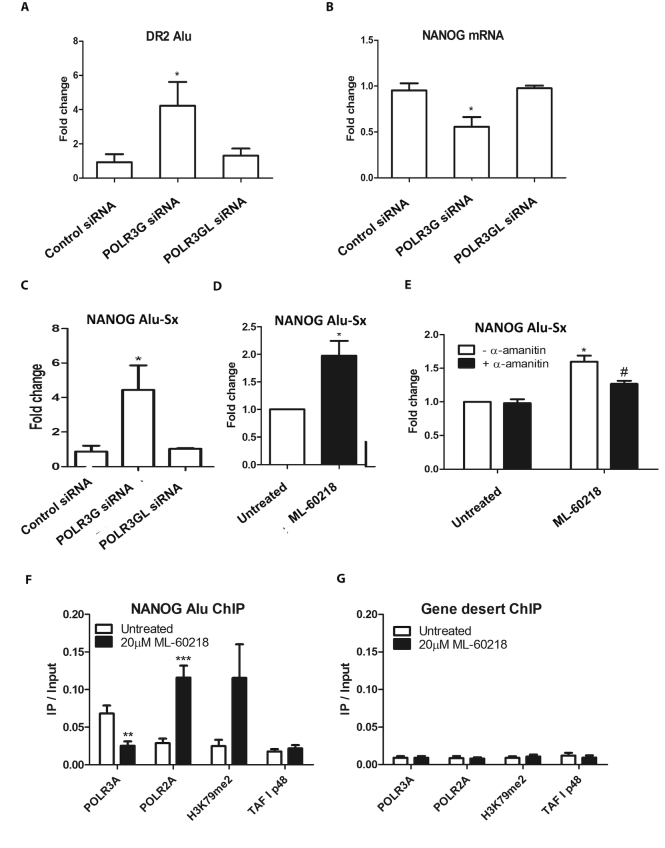
Effects of POLR3G RNAi and ML-60218 on DR2 Alu. (**A**) Mean relative changes in normalized expression of DR2 Alu RNA in PC-3 cells harvested 48 hrs after transfection with control siRNA or siRNAs targeting POLR3G or POLR3GL, as determined by RT-qPCR in three independent experiments. (**B**) Mean relative changes in normalized expression of NANOG mRNA in PC-3 cells harvested 48 h after transfection with control siRNA or siRNAs targeting POLR3G or POLR3GL, as determined by RT-qPCR in three independent experiments. (**C**) Mean relative changes in normalized expression of NANOG-Alu-Sx RNA in PC-3 cells harvested 48 h after transfection with control siRNA or siRNAs targeting POLR3G or POLR3GL, as determined by RT-qPCR in three independent experiments. (**D**) Mean relative changes in normalized expression of NANOG-Alu-Sx RNA in PC-3 cells harvested 48 h after treatment with or without 20 μM ML-60218, as determined by RT-qPCR in three independent experiments. (**E**) Mean relative changes in normalized expression of NANOG-Alu-Sx RNA in PC-3 cells harvested 48 h after treatment with or without 20μM ML-60218, as determined by RT-qPCR in 3 independent experiments. Black bars indicate samples that were exposed to 10μg/ml α-amanitin for 4 h prior to harvesting. (**F**) Mean ratios relative to input of NANOG-Alu-Sx DNA immunoprecipitated using antibodies against POLR3A, POLR2A, H3K79me2 and TAF_I_48 (negative control) with chromatin from PC-3 cells harvested 48 h after treatment with or without 20 μM ML-60218, as determined by ChIP-qPCR in six independent experiments. (**G**) Mean ratios relative to input of gene desert DNA immunoprecipitated using antibodies against POLR3A, POLR2A, H3K79me2 and TAF_I_48 (negative control) with chromatin from PC-3 cells harvested 48 h after treatment with or without 20 μM ML-60218, as determined by ChIP-qPCR in 6 independent experiments. * indicates *P* < 0.05 relative to control by *t*-test; ** indicates *P* < 0.01; *** indicates *P* < 0.005. # indicates *P* < 0.05 by *t*-test for +α-amanitin relative to –α-amanitin. Error bars represent S.E.M.

Additional templates transcribed by pol III include the Alu short interspersed nuclear elements (SINEs). A subset of the family, termed DR2, produces transcripts that are processed by DICER into small repeat-induced RNAs (riRNAs) that target specific mRNAs for degradation (18,42). An important target that is regulated in this way is NANOG, which controls stem cell regeneration (18,42). Overexpression of DR2 Alus in hESC suppresses NANOG and stem cell proliferation, whereas depletion of DR2 Alu transcripts impairs differentiation (18,42). We therefore investigated whether the distinct effects of POLR3G and POLR3GL on differentiation and proliferation might involve DR2 Alus. In support of this, DR2 Alu RNA levels increase when POLR3G is depleted by RNAi, but are unaffected by comparable depletion of POLR3GL (Figure 3A). NANOG mRNA responds in a reciprocal manner (Figure 3B), consistent with its suppression by DR2 Alu riRNA (18,42). A member of the DR2 Alu family lies ∼6kb upstream of the NANOG gene and has been dubbed NANOG-Alu-Sx; it is transcribed to produce RNAs that are processed into riRNAs with high complementarity to a region in the 3′-UTR of NANOG mRNA that mediates its turnover (18). As with the DR2 Alu family in general (Figure 3A), expression of NANOG-Alu-Sx is unchanged by depletion of POLR3GL but increases following RNAi of POLR3G (Figure 3C). These data suggest that POLR3G selectively exerts an inhibitory influence on expression of DR2 Alu transcripts. This suppressive effect might in principle involve obstruction of the promoter or transcribed region. Alternatively, the chromatin landscape might become more conducive to DR2 Alu transcription as proliferation slows and differentiation commences following depletion of POLR3G.

Because ML-60218 causes POLR3G levels to fall (Figure 2E), it might allow induction of NANOG-Alu-Sx. Indeed, NANOG-Alu-Sx expression increases in response to ML-60218 (Figure 3D). As this treatment inhibits pol III, we postulated that the induced transcripts are synthesized by pol II. Most RNAs that contain Alu sequences are thought to be pol II products, as the pol III promoter is weak and/or epigenetically repressed in most Alu copies (43–49). In some SINEs, pol III conducts basal transcription, but is replaced by pol II to allow elevated expression in response to specific stimuli (50). In support of this scenario, selective pol II inhibition with low dose α-amanitin suppresses the induction of NANOG-Alu-Sx in response to ML-60218, whilst having no effect on its basal expression in untreated cells (Figure 3E). ChIP confirmed the recruitment of pol II (POLR2A catalytic subunit) to NANOG-Alu-Sx in the presence of ML-60218, whilst the signal from pol III (POLR3A catalytic subunit) decreases in parallel (Figure 3F). ChIP specificity was demonstrated using a ‘gene-desert’ locus free of recognizable genes (Figure 3G). Pol II-transcribed regions can be identified by methylation of histone H3 on lysine 79 (H3K79), a modification not associated with pol III activity (21). ChIP with antibody specific for methylated H3K79 showed induction of this mark at NANOG-Alu-Sx following treatment with ML-60218. This provides evidence that the pol II recruited to this site is active, as H3K79 is methylated specifically by the pol II elongation complex during transcription (51). These data can explain the expression of NANOG-Alu-Sx when pol III is inhibited using ML-60218. We conclude that pol II can replace pol III to transcribe NANOG-Alu-Sx when POLR3G is depleted by RNAi or ML-60218 treatment.

**Figure 4.**
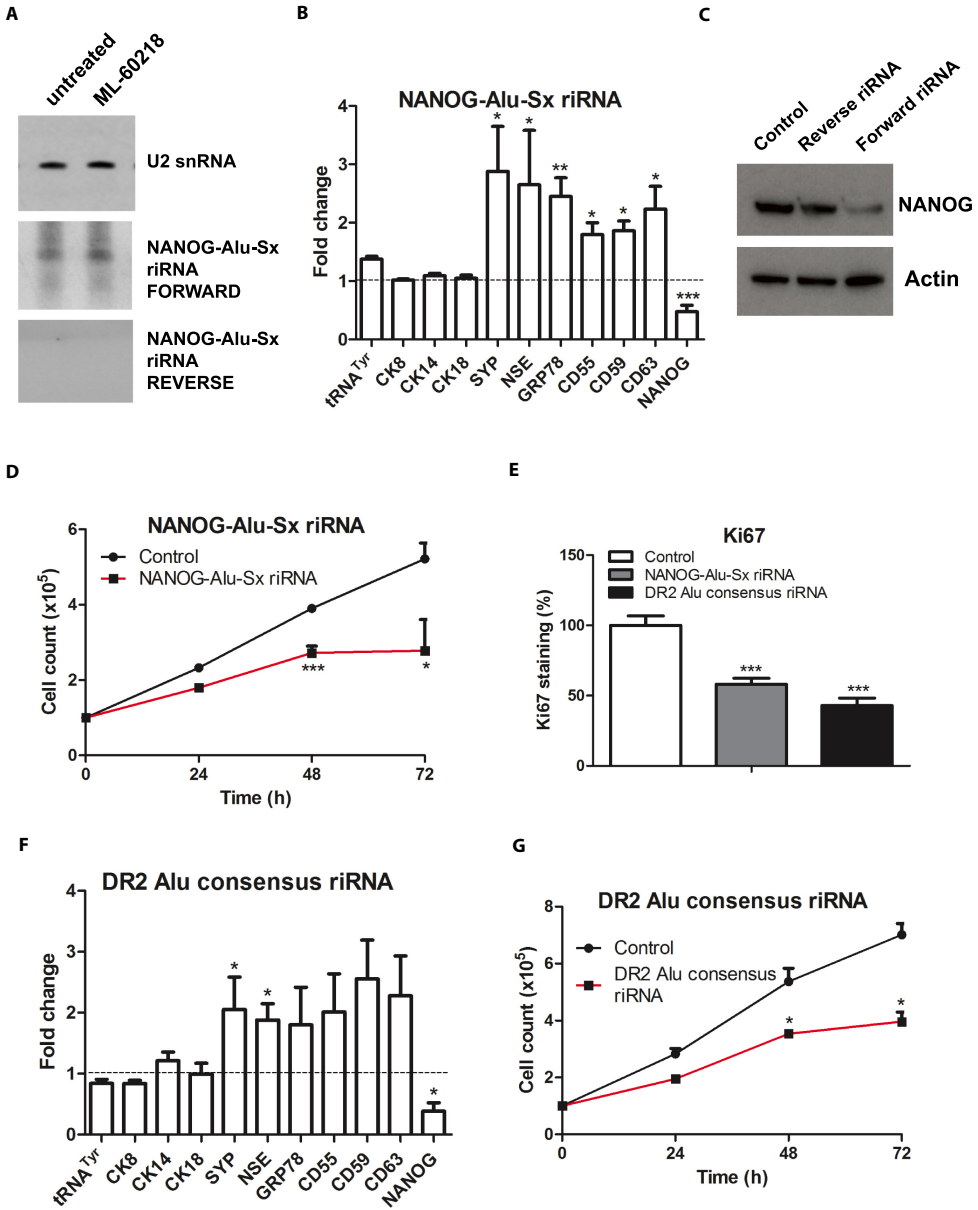
Effects of DR2 Alu RNA on differentiation and proliferation of PC-3 cells. (**A**) Northern blot of RNA harvested from PC-3 cells after 48 h treatment with or without 20 μM ML-60218 and hybridized with probes for U2 snRNA (pol II-transcribed control) and each strand of NANOG-Alu-Sx RNA. (**B**) Means of the relative changes in normalized expression of unprocessed pre-tRNA^Tyr^ and mRNAs encoding CK8, CK14, CK18, SYP, NSE, GRP78, CD55, CD59, CD63 and NANOG, as determined by RT-qPCR in 3–5 independent experiments, in PC-3 cells harvested 48 hrs after transfection with a 33nt synthetic RNA corresponding to part of the NANOG-Alu-Sx sequence. (**C**) Western blot of NANOG and actin protein expression in PC-3 cells 48 h after mock transfection (control) or transfection with a 33nt synthetic RNA corresponding to forward or reverse strands of part of the NANOG-Alu-Sx sequence. (**D**) Means of PC-3 cell numbers over 3 days following transfection with a 33nt synthetic RNA corresponding to part of the NANOG-Alu-Sx sequence, as indicated, in 4 independent experiments. (**E**) Means of the percentage of PC-3 cells staining positively for Ki67 proliferation marker 48 h after mock transfection or transfection with a 33nt synthetic RNA corresponding to part of the NANOG-Alu-Sx sequence or a 56nt synthetic RNA corresponding to part of the DR2 Alu consensus sequence. (**F**) Means of the relative changes in normalized expression of unprocessed pre-tRNA^Tyr^ and mRNAs encoding CK8, CK14, CK18, SYP, NSE, GRP78, CD55, CD59, CD63 and NANOG, as determined by RT-qPCR in three independent experiments, in PC-3 cells harvested 48 h after transfection with a 56nt synthetic RNA corresponding to part of the DR2 Alu consensus sequence. (**G**) Means of PC-3 cell numbers over 3 days following transfection with a 56nt synthetic RNA corresponding to part of the DR2 Alu consensus sequence, as indicated, in three independent experiments. * indicates *P* < 0.05 relative to control by *t*-test; ** indicates *P* < 0.01; *** indicates *P* < 0.005. Error bars represent S.E.M.

NANOG-Alu-Sx RNA can be processed into short riRNA that targets NANOG mRNA for degradation (18). Northern blots with strand-specific NANOG-Alu-Sx probes detected an riRNA of ∼105 nts that is complementary to the 3′-UTR of NANOG mRNA (Figure 4A). Transfection of a 33nt synthetic RNA corresponding to part of this riRNA suppressed NANOG and induced expression of differentiation markers (Figure 4B and C). These changes in gene expression were not seen when the opposite strand from the same region was transfected (Figure 4C and Supplementary Figure S4). In addition, the NANOG-Alu-Sx RNA fragment reduced cell proliferation (Figure 4D and E). Synthetic RNAs corresponding to the DR2 Alu consensus sequence can also suppress NANOG mRNA in PC-3 cells, as shown previously in hESC (42); this is accompanied by induction of NE markers and reduced proliferation (Figure 4E–G). It therefore seems probable that riRNAs derived from multiple Alu loci can influence the balance between proliferation and differentiation in prostate cancer cells. Nevertheless, a specific locus, NANOG-Alu-Sx, is sufficient to generate riRNA that causes NANOG mRNA degradation to trigger differentiation. Production of this riRNA is suppressed by POLR3G, allowing proliferation and self-renewal.

### Prostate cancer cells show enhanced sensitivity to ML-60218

**Figure 5.**
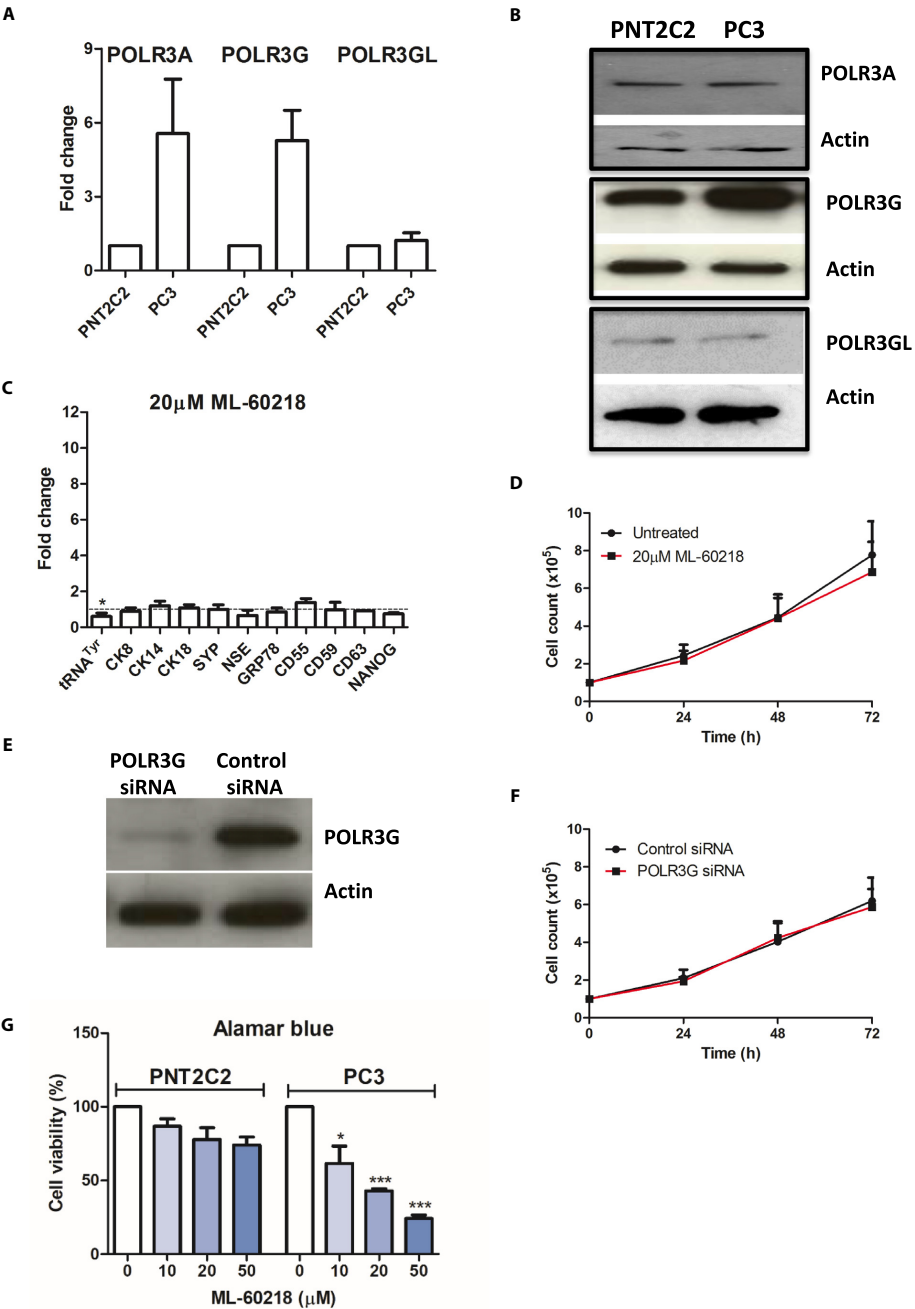
Benign PNT2C2 prostate cells express less POLR3G and are less sensitive to ML-60218 than PC-3 cells. (**A**) Normalized relative levels of POLR3A, POLR3G and POLR3GL mRNAs in PNT2C2 and PC-3 cells, as determined by RT-qPCR in two independent experiments. (**B**) Western blot of POLR3A, POLR3G, POLR3GL and actin proteins in PC-3 and PNT2C2 cells. (**C**) Means of the relative changes in normalized expression of unprocessed pre-tRNA^Tyr^ and mRNAs encoding CK8, CK14, CK18, SYP, NSE, GRP78, CD55, CD59, CD63 and NANOG in PNT2C2 cells harvested 48 hrs after treatment with or without 20μM ML-60218, as determined by RT-qPCR in three independent experiments. (**D**) Means of PNT2C2 cell numbers over 3 days in presence or absence of 20μM ML-60218, in three independent experiments. (**E**) Western blot of POLR3G and actin in PNT2C2 cells harvested 48 h after transfection with control or POLR3G-targeting siRNAs. (**F**) Means of PNT2C2 cell numbers over 3 days following transfection with control or POLR3G-targeting siRNAs, as indicated, in three independent experiments. (**G**) Means of the percentage of viable PNT2C2 and PC-3 cells, as determined using Alamar blue, after 2 days exposure to the indicated concentrations of ML-60218, in three independent experiments. * indicates *P* < 0.05 relative to control by *t*-test; *** indicates *P* < 0.005. Error bars represent S.E.M.

We compared the PC-3 cancer cell line with PNT2C2, a non-tumourigenic line of immortalized healthy prostate epithelium (52). The relative expression of mRNA encoding POLR3A is higher in PC3 than in PNT2C2, but this difference is not reflected at the protein level (Figure 5A and B). However, both mRNA and protein of POLR3G are elevated in the cancer cells relative to the untransformed cells. In contrast, levels of POLR3GL mRNA and protein are similar between these prostate lines (Figure 5A, B and Supplementary Figure S5). These observations are consistent with previous evidence that cancer cells overexpress POLR3G selectively (2,8). Although ML-60218 inhibits pol III-dependent transcription in PNT2C2 cells, it does not induce the differentiation markers that are induced in PC-3 cells (Figure 5C). Furthermore, exposure for three days to 20μM ML-60218 has minimal effect on the rapid proliferation of PNT2C2 cells, in contrast to PC-3 (Figure 5D). The same is true for POLR3G depletion by RNAi (Figure 5E and F). The Alamar Blue assay, a measure of cellular health and viability (53), also revealed far less sensitivity of PNT2C2 to ML-60218 compared with PC-3 (Figure 5G). This was confirmed by staining with Annexin V and propidium iodide, which showed that only the PC-3 cells display increased apoptosis under these conditions (Supplementary Figure S6). The relative tolerance of PNT2C2 cells to ML-60218 does not reflect a compromised apoptotic pathway, as a strong response is elicited by the alkaloid staurosporine (Supplementary Figure S6). Thus, PC-3 cancer cells are significantly more sensitive to pol III inhibition than untransformed PNT2C2 cells.

**Figure 6.**
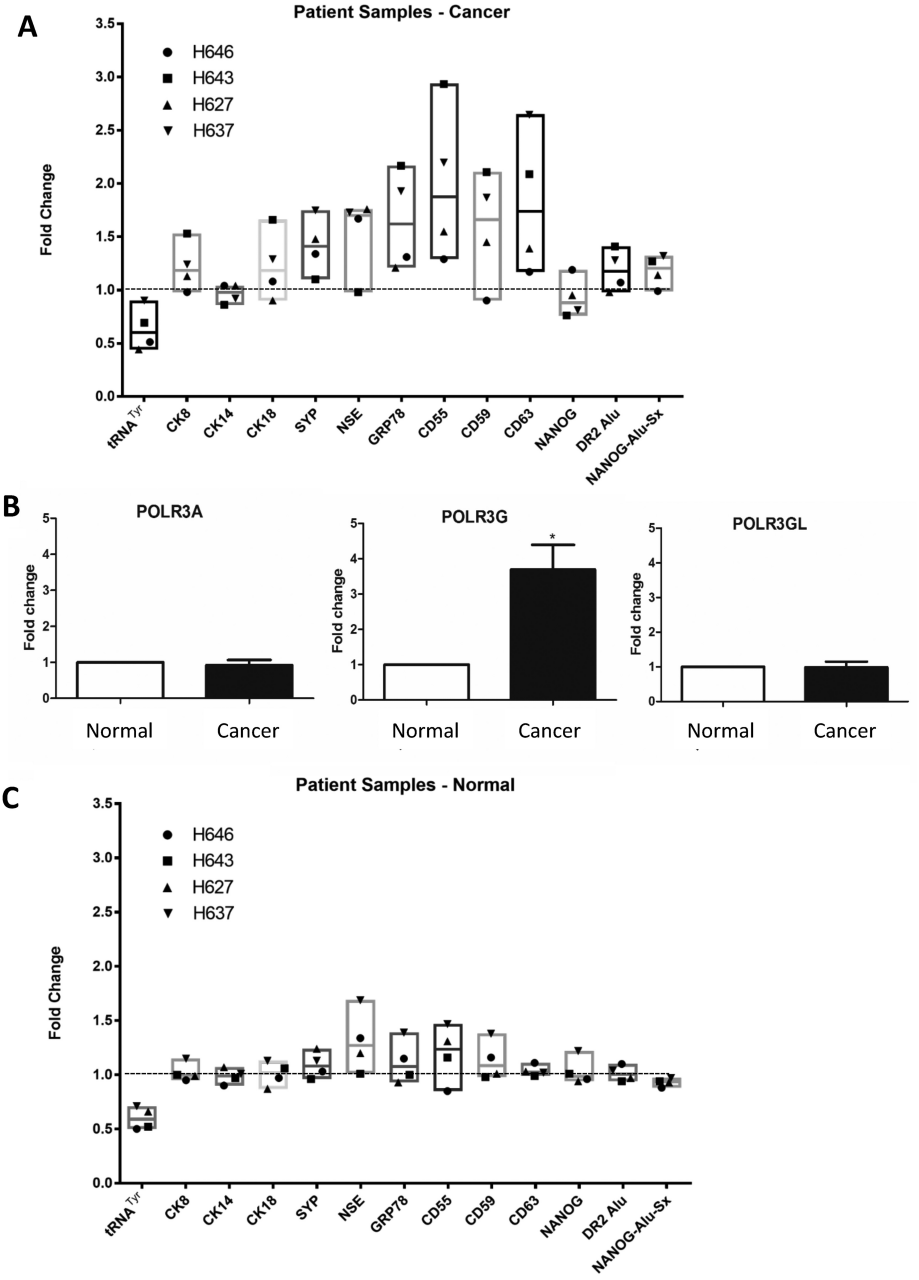
Primary cells from prostate tumours differentiate in response to ML-60218. (**A**) Relative changes in normalized expression of the indicated transcripts, as determined by RT-qPCR, when tumour cells from patients H646, H643, H627 and H637 were treated for 48 h with or without 20 μM ML-60218. Means are indicated by horizontal lines and values for each patient are indicated by symbols. (**B**) Normalized relative levels of POLR3A, POLR3G and POLR3GL mRNAs in normal and cancer cells from patients H646, H643, H627 and H637, as determined by RT-qPCR. * indicates *P* < 0.05 relative to normal by *t*-test. Error bars represent S.E.M. (**C**) Relative changes in expression of the indicated transcripts, as determined by RT-qPCR, when cells from non-cancerous tumour-adjacent cells from patients H646, H643, H627 and H637 were treated for 48 h with or without 20 μM ML-60218. Means are indicated by horizontal lines and values for each patient are indicated by symbols.

Primary cells were cultured from the tumours of four patients with Gleason grade 7 cancers, obtained during radical prostatectomy. There was heterogeneity between patients in the responses to treatment for 48 h with 20 μM ML-60218, but pre-tRNA^Tyr^ decreased by 36% on average, whereas mRNA encoding the basal cell marker CK14 showed minimal change (Figure 6A). In tumours from two of the four patients (H643 and H637), ML-60218 induced DR2 Alu SINEs, including NANOG-Alu-Sx, and this was accompanied by suppression of NANOG mRNA. Alu induction and NANOG repression was weaker than in PC-3 cells, perhaps because the tumour biopsies included untransformed contaminating cells. Nevertheless, these tumours showed induction of CK8, CK18, GRP78, CD55, CD59 and CD63 mRNAs (Figure 6A). In general, SYP and NSE mRNAs were also induced, but this was not the case for H643. This suggests that ML-60218 triggered differentiation of tumour H643 towards a luminal phenotype (demonstrated by CK8 and CK18 markers) without the accompanying NE features indicated by SYP and NSE. In contrast, two tumours without NANOG suppression (H646 and H627) still showed induction of SYP and NSE, suggesting alternative pathways towards NE properties. Thus, the secondary transcriptomic changes caused by ML-60218 vary between patients. This is expected, given the individuality of genotype and molecular lesions in these cancers (54). Furthermore, the degree of cell differentiation varied between regions of each tumour, adding to heterogeneity between samples.

At the same time as these tumours were removed, adjacent healthy material was recovered from the same organs. The samples of matched non-cancerous prostate were cultured and treated in parallel to the tumours from the same patients. Whereas POLR3A and POLR3GL were expressed at comparable levels, POLR3G expression was on average 3.7-fold higher in tumours relative to adjacent prostate samples from the same individuals (Figure 6B). There was again heterogeneity between patients, with H627 showing much weaker overexpression of POLR3G than the others (Supplementary Figure S7). In these primary non-cancerous cells, ML-60218 inhibited pre-tRNA^Tyr^ by 40%, comparable to the matched cancer cells (36% inhibition), but had minimal effect on DR2 Alu, NANOG, CK8 or CK14 expression (Figure 6C). NSE, GRP78, CD55 and CD59 sometimes increased, but for each patient the response of the non-cancerous prostate sample was weaker than that of the tumour sample. Thus, as with the cell line models, normal primary cells differentiate less readily than matched cancer cells in response to ML-60218.

**Figure 7.**
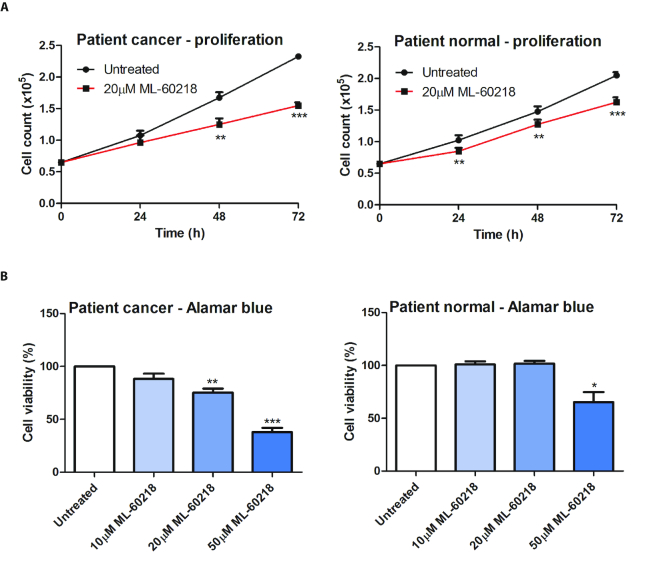
Primary cells from prostate tumours are more sensitive to ML-60218 than adjacent non-cancerous cells from the same tissues. (**A**) Means of cell numbers from tumours (left) and non-cancerous tumour-adjacent samples (right) from patients H646, H643, H627 and H637 during culture for 3 days in the presence or absence of 20 μM ML-60218. (**B**) Means of the number of viable cells, as determined using alamar blue, after 2 days of exposure of tumour samples (left) and non-cancerous tumour-adjacent samples (right) from patients H646, H643, H627 and H637 to the indicated concentrations of ML-60218, in three independent experiments. * indicates *P* < 0.05 relative to control by *t*-test; ** indicates *P* < 0.01; *** indicates *P* < 0.005. Error bars represent S.E.M.

The pol III inhibitor slowed proliferation of both normal and cancer cells from the four patients (Figure 7A), although cells from the tumours were significantly more sensitive (*P* < 0.01 after 72 h). However, the viability of the normal cells was almost unperturbed after 48 h exposure to 20 μM ML-60218, whereas that of the tumour cells was suppressed by 25% (Figure 7B). Higher concentrations of ML-60218 do affect viability of the normal cells, but still have a greater impact on the cancer cells. These data suggest that oncogenic transformation enhances the sensitivity of prostate cells to pol III inhibition, which may provide a useful vulnerability.

### ML-60218 can deplete tumour initiating cells

**Figure 8.**
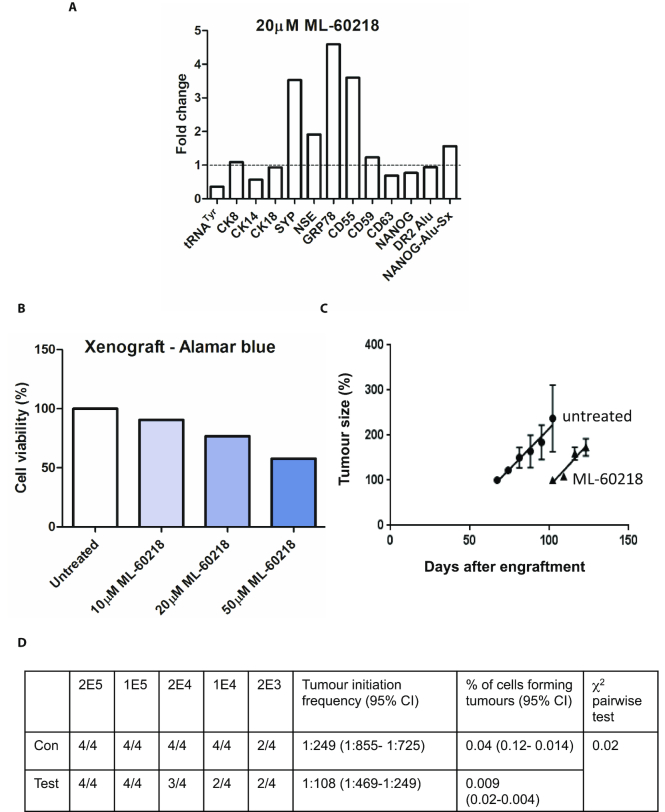
Transient exposure to ML-60218 can diminish tumour initiation *in vivo*. (**A**) Relative changes in normalized expression of the indicated transcripts, as determined by RT-qPCR, when dissociated tumour cells from CRPC patient H455 were treated for 48 hrs with or without 20 μM ML-60218. (**B**) Percentages of viable cells, as determined using alamar blue, after 2 days exposure of H455 tumour samples to the indicated concentrations of ML-60218. (**C**) Growth rates of tumours, following injection of 10^5^ H455 cells pre-treated for 48 hrs with (triangles) or without (circles) 20 μM ML-60218 (*n* = 4 mice per group). The relative value of 100% refers to a volume of ∼50 mm^3^. (**D**) Numbers of mice developing tumours after groups of four mice were injected subcutaneously with the indicated numbers of viable H455 tumour cells after 48 h pre-treatment without (con) or with (test) 20 μM ML-60218. Calculated tumour initiation frequencies and percentages of tumour-forming cells are shown for treatment and control arms.

The cancer stem cell (CSC) hypothesis posits that tumours contain tiny populations of long-lived, pluripotent stem cells with the capacity for self-renewal and differentiation into the various lineages of the tumour (55,56). CSC can survive radiation or chemotherapy and then drive disease recurrence leading to relapse after therapy (56). The frequency of such tumour initiating cells in a heterogeneous population can be estimated by engrafting into mice at limiting dilutions (23,57). This xenograft assay was conducted using androgen-independent tumour cells derived from a patient with CRPC (22). Treatment for 48 h in culture with 20 μM ML-60218 again induced differentiation markers such as SYP, NSE and GRP78 (Figure 8A). As with the cancer cells from other patients, viability was compromised, in this case by ∼23% (Figure 8B). This loss was taken into account when the cells were engrafted into mice, so that control and treatment arms received equal numbers of viable human cells. Dilution series were engrafted into mice and tumour development monitored. Exposure to ML-60218 was found to delay tumourigenesis significantly; for example, a mean latency period of 75 days after injection of 10^5^ viable untreated cells was extended to 100 days when the cells were pre-treated for 48 h with 20 μM ML-60218 (Figure 8C). Furthermore, pairwise tests showed a significant decrease in the frequency of tumour initiation (*P* < 0.05) and that the percentage of cells forming tumours was reduced by ∼4-fold (Figure 8D). Once initiated, the tumours grew at similar rates in the absence of drug, in contrast to the impaired proliferation observed in its presence (Figure 7A). Nevertheless, the data indicate that transient exposure to ML-60218 can have selective and enduring effects on tumour initiating activity, consistent with CSC depletion.

## DISCUSSION

**Figure 9.**
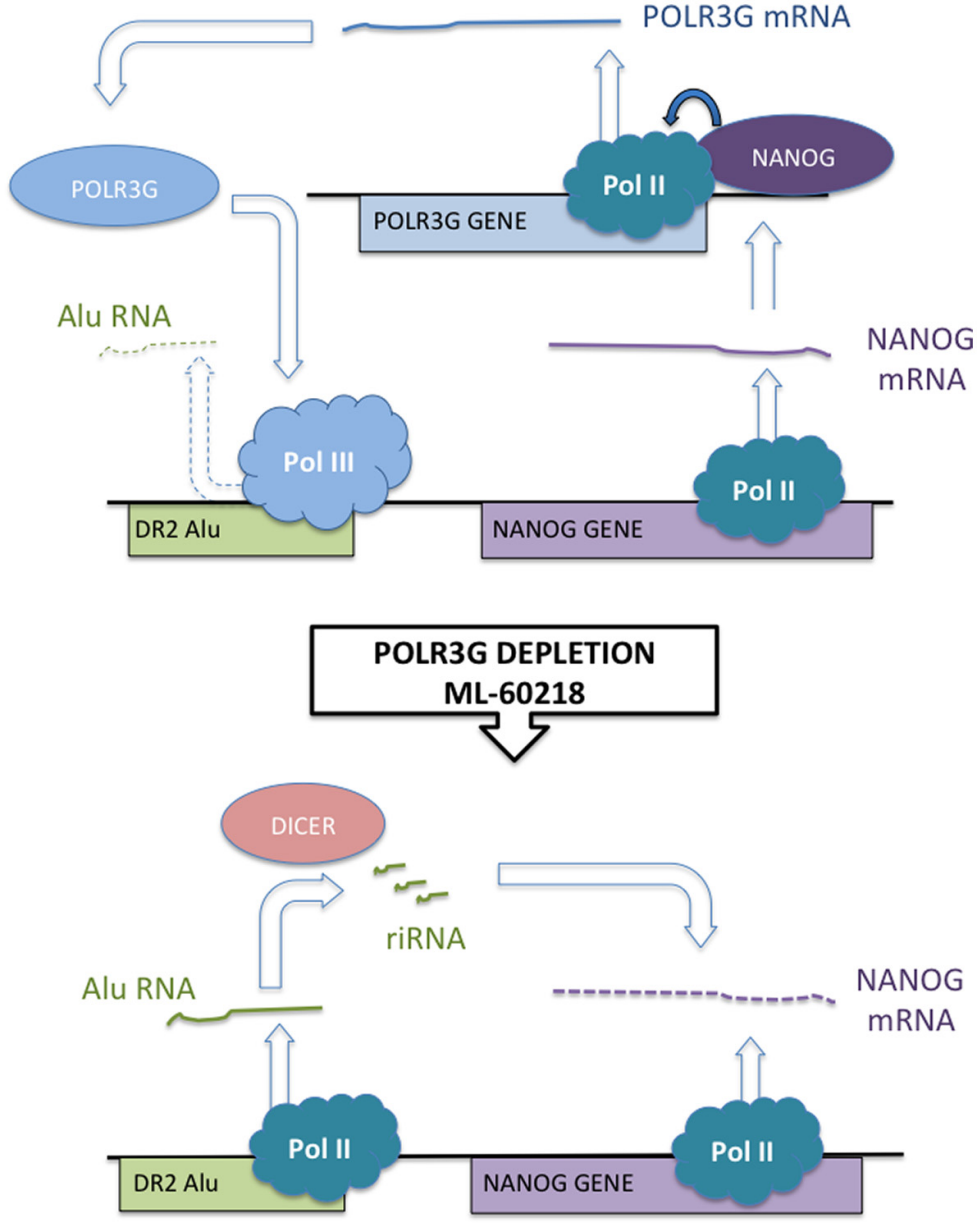
Model to explain the observed response of prostate cancer cells to POLR3G depletion following RNAi or ML-60218 treatment. Undifferentiated cells express NANOG, which has been shown to bind the promoter of the POLR3G gene and stimulate its expression. Pol III associated with POLR3G binds DR2 Alu SINEs but does not transcribe them efficiently. When POLR3G is depleted by RNAi or treatment with ML-60218, levels of pol III at DR2 Alu SINEs decrease whereas levels of pol II increase; transcription of these loci rises, generating transcripts that are processed by dicer into repeat-induced small RNAs (riRNA) that target NANOG mRNA for AGO-mediated degradation; falling levels of NANOG trigger differentiation.

Many studies have described links between pol III and cancer (e.g. (8,58–76)}; reviewed by (77–81)). Most relevant to the current work are reports that POLR3G overexpression can have oncogenic effects in cultured cells and mice (2,6). Conversely, RNAi of POLR3G causes differentiation of hESC (5,7). We found that prostate cancer cells also differentiate when depleted of POLR3G, either by RNAi or by treatment with ML-60218. The resultant differentiation is likely to involve multiple changes in transcript expression, but can be mimicked simply by overexpressing riRNA that is suppressed by POLR3G and in turn controls levels of the pluripotency factor NANOG (Figure 4). POLR3G expression may be amplified by positive feedback, as NANOG binds and activates the gene encoding POLR3G (5), stabilizing the undifferentiated state (Figure 9). Such a scenario is consistent with evidence that hESC overexpressing POLR3G are resistant to differentiation signals (5,7).

RiRNAs that target NANOG are produced by DICER-dependent processing of transcripts containing DR2 Alu sequences (18,42). Such transcripts are made predominantly by pol III in hESC (18,42), but Alu SINEs are also commonly expressed by pol II as part of larger transcripts (47). These properties reflect SINE generation by retrotransposition and random insertion, often near or within pol II-transcribed genomic regions. Both pol II and pol III can be detected at the NANOG-Alu-Sx and expression increases when pol II replaces pol III (Figure 3). Precedent for such behaviour comes from the B1-X35S subfamily of B1 SINEs, the rodent equivalent of Alu, where pol III mediates basal transcription but can be replaced by pol II to raise expression (50). This property allows B1-X35S to be induced by aryl hydrocarbon receptor and it is noteworthy that NANOG-Alu-Sx also contains a functional binding site for this activator (18). In addition, NANOG-Alu-Sx contains the DR2 motif that mediates Alu induction by retinoic acid receptors (18,42). Such response elements allow regulatory stimuli to control transcription of Alu subsets. Indeed, expression of individual Alu loci varies considerably between cell types and growth conditions (82).

Depletion of POLR3G impacts cell behaviour in ways that are not seen when POLR3GL is depleted. This is consistent with the finding that overexpression of POLR3G enhances the ability of partially transformed human fibroblasts to form anchorage-independent colonies, an effect not seen with POLR3GL (2). A simple hypothesis is that these paralogous subunits mediate production of transcriptomes that differ in some crucial way(s), although they probably overlap substantially. Indeed, patterns of tRNA expression vary markedly between proliferating and differentiating cells (71). Although clear differences were not seen in vitro between purified pol III containing POLR3G or POLR3GL, levels of some pol III transcripts were selectively increased in fibroblasts by overexpression of POLR3G (2). For example, tRNA_i_^Met^ levels increased when POLR3G was overexpressed, but tRNA^Glu^ did not (2). Depletion of POLR3G from hESC also had differential effects on individual pol III products (5). A role in target discrimination is consistent with the location of POLR3G/GL in a module that interfaces between the catalytic core of pol III and promoter-bound transcription factors (83–85). Solvent exposed residues (85) may allow contacts with regulators that distinguish between the paralogues, potentially mediating differential recruitment and/or activity of pol III. When ChIP-Seq was used to compare binding sites in fibroblasts, the large majority of loci occupied by POLR3G were also occupied by POLR3GL and vice versa, but relative enrichment for one or other paralogue was observed at a subset of genes (3). Consistent with this, both paralogues can be detected at NANOG-Alu-Sx in PC3 cells (Supplementary Figure S8). Nevertheless, expression of DR2 Alu RNAs in PC3 cells responds to depletion of POLR3G, but not POLR3GL (Figure 3A). Examples have been detected where loci are bound by pol III but apparently not transcribed (46). A parsimonious model is that pol III activity at DR2 Alus is sensitive to whether it contains POLR3G or POLR3GL, perhaps due to selective interaction(s) with regulators. Expression is enhanced when pol III is replaced by pol II, which can respond more strongly to activators, a switch that can be triggered by selective depletion of POLR3G.

A variety of tRNA fragments have been shown to influence cell proliferation and/or viability (86). These include tRFs and SHOT-RNAs, which can stimulate proliferation in prostate cancer cells (87,88). However, rates of tRNA synthesis do not control levels of tRNA fragments, which are dictated by the cleavage reactions and do not correlate with expression of full-length tRNA (86,88,89). It therefore seems unlikely that tRNA fragments contribute substantially to the observed responses to POLR3G depletion, although their involvement cannot be excluded. Other pol III products might also be involved, such as NDM29 and/or 45A ncRNAs (58,59). Furthermore, the secondary effects downstream of inhibiting any RNA polymerase are likely to be diverse. Nevertheless, our ability to reproduce much of the response using synthetic versions of Alu riRNAs that are induced by ML-60218 suggests that Alu-mediated effects can provide a key component of the phenotype elicited when pol III is inhibited in prostate cells.

There is a clinical need for more effective interventions against CRPC (15). As well-differentiated tumours generally have better prognosis and POLR3G depletion triggers differentiation of hESC, we hoped it might have similar effects in a model of advanced disease. Indeed, RNAi of POLR3G reduces proliferation and triggers differentiation and/or death of PC-3 cells, whereas benign PNT2C2 prostate cells are less sensitive, despite lower POLR3G expression. This differential response is reproduced using ML-60218, a cell-permeable small molecule that inhibits pol III and triggers selective depletion of POLR3G. Although the possibility of off-target effects cannot be excluded, the fact that differentiation and proliferation respond in the same way to ML-60218 as to siRNAs against POLR3A and POLR3G provides confidence that the phenotype is mediated through pol III.

The response involves a specific decrease in expression of the pluripotency marker NANOG, which suggests that CSC are depleted. Support for this comes from measurements of tumour initiation frequency in vivo using cells derived from a CRPC patient. Limiting dilutions allowed us to estimate that 0.04% of patient-derived cells were capable of seeding a new tumour in our xenograft model (Figure 8D). This subpopulation with CSC activity is considered to be responsible for disease recurrence and relapse after therapy (13,56). Ex vivo exposure to ML-60218 reduced tumour initiation frequency ∼4-fold when the cells were grafted back into mice. These data provide functional evidence that ML-60218 is not only effective against the bulk population of tumour cells from a CRPC patient, but can also impair the initiation of new tumours by CSCs that carry the greatest threat to prognosis.

In other mammalian cell types, specific tRNAs have been found to promote migration and invasion in culture, as well as metastasis in mice (73–75). For example, migration of fibroblasts and melanoma cells can be stimulated by tRNA_i_^Met^ (73,74). As tRNA_i_^Met^ expression in PC-3 cells is reduced by ML-60218 (Figure 2G), we tested whether their invasive behaviour is affected. Indeed, the ability of PC-3 cells to invade matrigel is suppressed significantly by treatment with ML-60218 (Supplementary Figure S9). In breast cancer cells, invasion and metastasis is promoted by tRNA^Glu^_UUC_ and tRNA^Arg^_CCG_ (75). We found that PC-3 cells express several genes encoding these tRNAs and that levels of their primary transcripts decrease significantly in response to ML-60218 (Supplementary Figure S10). These data support the possibility that partial pol III inhibition might reduce cancer spread. Effects on protein synthesis and cell growth are also likely (77,79).

Since pol III is an essential enzyme, its targeting raises concerns about general toxicity. However, sensitivity varies according to cell type. This was clearly illustrated in zebrafish by a hypomorphic mutation in the POLR3B subunit of pol III (90). Despite global decreases in tRNA levels to ∼40% of wild-type, defects only appeared in highly proliferative tissues, such as intestine and exocrine pancreas (90). Human pancreatic adenocarcinoma lines are more sensitive to ML-60218 than untransformed pancreatic epithelial cells (91). Similarly, the benign prostate lines PNT2C2 and BPH1 tolerate ML-60218 better than the PC-3 and DU145 prostate cancer cell lines (Figure 5 and Supplementary Figures S6 and S11) and primary cells from tumours are significantly more sensitive than non-cancerous cells from elsewhere in the same prostates (Figure 7). Although these adjacent cells appear normal, they may carry some of the genetic and/or epigenetic changes responsible for the tumour that necessitated prostatectomy (13). We therefore also tested primary prostate epithelial cells from an organ donor, presumed to be cancer-free. Two days treatment with 20 μM ML-60218 had minimal effect on viability or expression of differentiation markers in these cells, despite efficiently suppressing pre-tRNA (Supplementary Figure S12). Collectively, the data point towards a therapeutic window, where cancer cells are more sensitive than healthy cells to depletion of pol III transcripts. The differential response may reflect levels of POLR3G, which is more abundant in tumours than in normal cells (Figure 6B). If so, the restricted expression and influence on pluripotency of POLR3G (2,4,5) suggest that pol III may provide a vulnerability where tumours can be attacked.

However, in considering pol III as a candidate drug target for prostate cancer, some potentially unfavourable features of the response to ML-60218 should be weighed against the benefits listed above. One concern is the induction of GRP78, a feature of aggressive variant prostate cancer with NE differentiation that is linked with poor prognosis (28,92). ML-60218 also induces CD55, which has been found to promote prostate cancer growth and survival (93). CD55 and GRP78 are themselves considered attractive targets for therapies directed against cell surface molecules (92,94); such approaches might be considered in combination with pol III inhibition. Another concern is that differentiation occurs towards an NE phenotype, which is generally associated with low survival (15,95). Indeed, transdifferentiation to an NE phenotype is an important mechanism of acquired resistance to treatments that target the androgen receptor (15,96). However, one tumour (H643) showed induction of luminal markers (CK8 and CK18) without the NE markers SYP and NSE (Figure 6). This distinct response was still associated with increased DR2 Alu RNA, suppression of NANOG mRNA and slowing of proliferation. It is unclear why this individual responded differently from the others, but he may indicate the existence of a subgroup that would benefit from the effects of treatment on tumour cell viability, differentiation and proliferation, without the potential hazards of NE induction. More extensive studies will be required to establish the prevalence of such a hypothetical subgroup or whether this case is an exceptional outlier.

## CONCLUSIONS

In summary, this study demonstrates that the inhibitory effect of POLR3G on cell differentiation, first discovered in hESC, is also a feature of prostate cancer cells. Although this property is not shared by its paralogue POLR3GL, it can be reproduced by depleting or inhibiting the pol III catalytic subunit POLR3A. We interpret this to mean that the influence of POLR3G on differentiation is mediated through pol III, as opposed to some hypothetical pol III-independent function. This influence can be explained, at least in part, by the ability of POLR3G to regulate expression of DR2 Alu SINEs, which themselves control levels of the pluripotency factor NANOG, through a post-transcriptional mechanism. Synthetic DR2 Alu RNA is sufficient to trigger differentiation in the PC-3 model. The cell-permeable small molecule ML-60218 inhibits pol III and causes differentiation, POLR3G depletion, and significant reductions in the proliferation, invasiveness, tumour-initiating activity and viability of prostate cancer cells. Untransformed cells appear to be less sensitive, raising the possibility of a therapeutic opportunity, if drugs can be developed with sufficient potency. Stratification might be necessary, through appropriate biomarkers, to identify tumours that differentiate towards luminal rather than NE phenotypes. It is also worth exploring how other cancer types respond to this approach.

## Supplementary Material

Supplementary Data
