# Supplementary material for: Effects on prostate cancer cells of targeting RNA polymerase III
Source: Nucleic Acids Res. 2019 Mar 1;47(8):3937–56. doi: 10.1093/nar/gkz128 (PMC6486637; doi:10.1093/nar/gkz128)
Supplement: Supplementary Data [file gkz128_supplemental_files.zip › Petrie et al. Revised Supplementary Figure Legends.docx]

**Supplementary Figure Legends**

**Supplementary Figure 1.**  POLR3A associates with both POLR3G and POLR3GL from PC-3 cells. Extracts of PC-3 cell proteins were immunoprecipitated using antiserum to POLR3A or matched preimmune serum and the pellets were probed by western blot for the presence of POLR3A, POLR3G and POLR3GL, as indicated.

**Supplementary Figure 2.** ML-60218 increases the appearance of dendritic processes amongst PC-3 cells. Representative images of PC-3 cells after 48hrs treatment without (upper) or with (lower) 20μM ML-60218.

**Supplementary Figure 3.**  Telomerase expression decreases when PC-3 cells are treated with ML-60218. Means of the relative changes in normalized expression of TERC RNA (lower) and mRNA encoding TERT protein (upper) in PC-3 cells harvested 48 hrs after treatment with or without 20µM ML-60218, as determined by RT-qPCR in 3 independent experiments. ** indicates *P* < 0.01 relative to control by *t*-test; *** indicates *P* < 0.005. Error bars represent s.e.m.

**Supplementary Figure 4.** Strand-specific effect of DR2 Alu RNA on mRNA expression in PC-3 cells. Means of the relative changes in normalized expression of unprocessed pre-tRNA^Tyr^ and mRNAs encoding CK8, CK14, CK18, SYP, NSE, GRP78, CD55, CD59, CD63 and NANOG, as determined by RT-qPCR in 3-5 independent experiments, in PC-3 cells harvested 48 hrs after transfection with a 33nt synthetic RNA corresponding to part of the NANOG-Alu-Sx sequence (FWD) or the complementary 33nt RNA (REV). * indicates *P* < 0.05 by *t*-test for FWD relative to REV riRNA; ** indicates *P* < 0.01; *** indicates *P* < 0.005. Error bars represent s.e.m.

**Supplementary Figure 5.**  Western blots comparing expression of POLR3G and POLR3GL in equal numbers of PC-3 and PNT2C2 cells. Purified recombinant protein is displayed for comparison.

**Supplementary Figure 6.**  ML-60218 induces cell death more strongly in PC-3 than in PNT2C2 cells. Representative images of FACS analyses of PC-3 (upper) and PNT2C2 cells stained with annexin V and propidium iodide after culture for 48hrs without treatment or with 20μM ML-60218, 50μM ML-60218 or 1μM staurosporine.

**Supplementary Figure 7.**  Comparison of pol III subunit expression in cancer and normal cells isolated in parallel from the same prostates. Relative levels of mRNAs encoding POLR3A **(A)**, POLR3G **(B)** and POLR3GL **(C)**, as determined by RT-qPCR and normalized to ARPP P0 mRNA, in matched normal and cancer prostate samples from patients H646, H643, H627 and H637, as indicated.

**Supplementary Figure 8.** Mean ratios relative to input of NANOG-Alu-Sx DNA (upper) or gene desert DNA (lower) immunoprecipitated using antibodies against POLR3A, POLR3G, POLR3GL and TAF_I_48 (negative control) with chromatin from PC-3 cells, as determined by ChIP-qPCR in 5 independent experiments.

**Supplementary Figure 9.** ML-60218 suppresses matrigel invasion by PC-3 cells. Means of the percentage of viable PC-3 cells that have invaded a matrigel matrix 48 hrs after treatment with or without 20μM ML-60218, as determined in 3 independent experiments. * indicates *P* < 0.05 relative to control by *t*-test. Error bars represent s.e.m.

**Supplementary Figure 10.** ML-60218 suppresses expression in PC-3 cells of tRNA genes implicated in invasion and metastasis. Means of the relative changes in normalized expression of pre-tRNA^Arg^-CCG (chr16.tRNA1 and chr6.tRNA114) and pre-tRNA^Glu^-UUC (chr2tRNA6, chr2.tRNA20 and chr1.tRNA84) in PC-3 cells harvested 48 hrs after treatment with or without 20μM ML-60218, as determined by RT-qPCR in 3-5 independent experiments. * indicates *P* < 0.05 relative to control by *t*-test; ** indicates *P* < 0.01. Error bars represent s.e.m.

**Supplementary Figure 11.** DU145 prostate cancer cells are more sensitive to ML-60218 than benign BPH1 prostate cells. (**A**) Mean numbers of benign BPH1 prostate epithelial cells, in 3 independent experiments, 48hrs after seeding 3x10^5^ cells in medium with 0μM, 10μM, 20μM or 50μM ML-60218. (**B**) Mean numbers of DU145 prostate cancer cells, in 3 independent experiments, 48hrs after seeding 3x10^5^ cells in medium with 0μM, 10μM, 20μM or 50μM ML-60218. (**C**) Means of the numbers of viable BPH1 cells, as determined using alamar blue, after 2 days exposure to 0μM, 10μM, 20μM or 50μM ML-60218 or 1μM staurosporine, in 3 independent experiments. (**D**) Means of the numbers of viable DU145 cancer cells, as determined using alamar blue, after 2 days exposure to 0μM, 10μM, 20μM or 50μM ML-60218 or 1μM staurosporine, in 3 independent experiments. * indicates *P* < 0.05 relative to control by *t*-test; ** indicates *P* < 0.01; *** indicates *P* < 0.005. Error bars represent s.e.m.

**Supplementary Figure 12.** Primary cells from healthy prostate epithelium are relatively unresponsive to ML-60218. (**A**) Means of the relative changes in normalized expression of unprocessed pre-tRNA^Tyr^ and mRNAs encoding CK8, CK14, CK18, SYP, NSE, GRP78, CD55, CD59, CD63 and NANOG in normal prostate epithelial cells harvested 48 hrs after treatment with or without 20μM ML-60218, as determined by RT-qPCR in 3 independent experiments. (**B**) Means of normal prostate epithelial cell numbers over 3 days with or without 20μM ML-60218, in 3 independent experiments. (**C**) Means of the percentage of viable primary cells from normal prostate epithelium, as determined using alamar blue, after 2 days exposure to the indicated concentrations of ML-60218, in 3 independent experiments. *** indicates *P* < 0.005 relative to control by *t*-test. Error bars represent s.e.m.
