# Supplementary material for: Effects on prostate cancer cells of targeting RNA polymerase III
Source: Nucleic Acids Res. 2019 Mar 1;47(8):3937–56. doi: 10.1093/nar/gkz128 (PMC6486637; doi:10.1093/nar/gkz128)

Supplementary Fig. 1

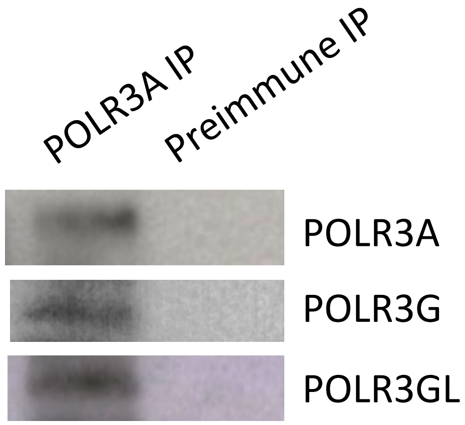

Supplementary Fig. 2

Untreated

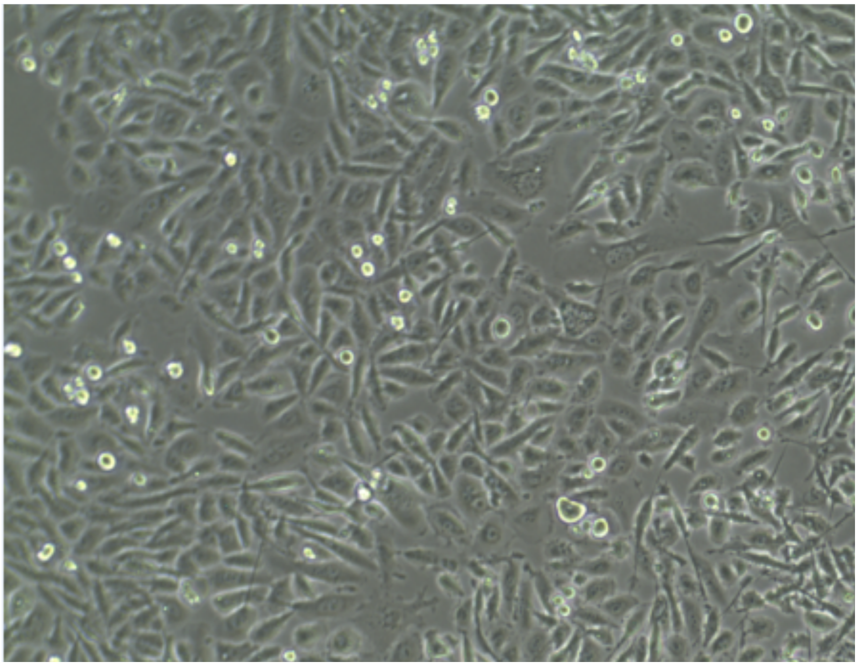

20 μM ML-60218

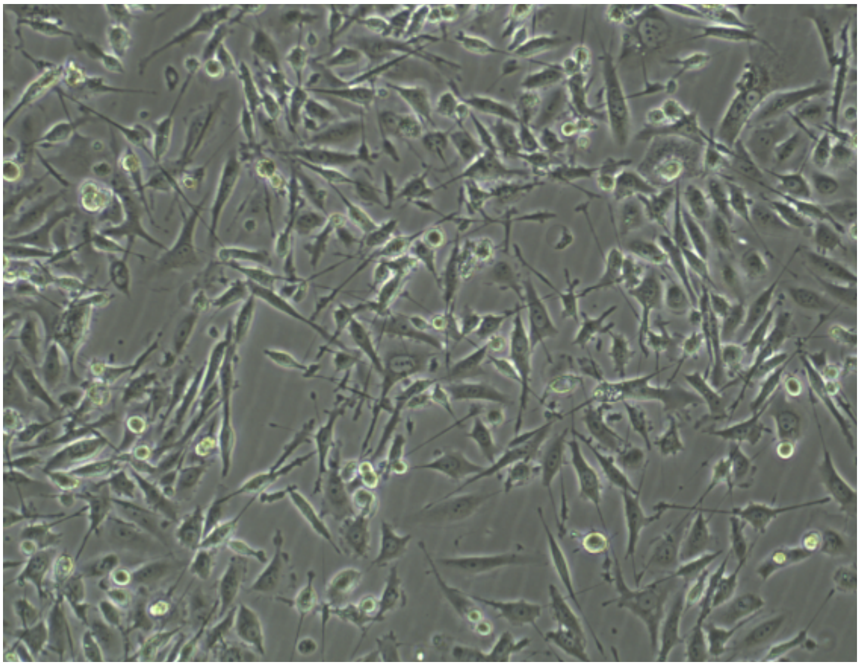

Supplementary Fig. 3

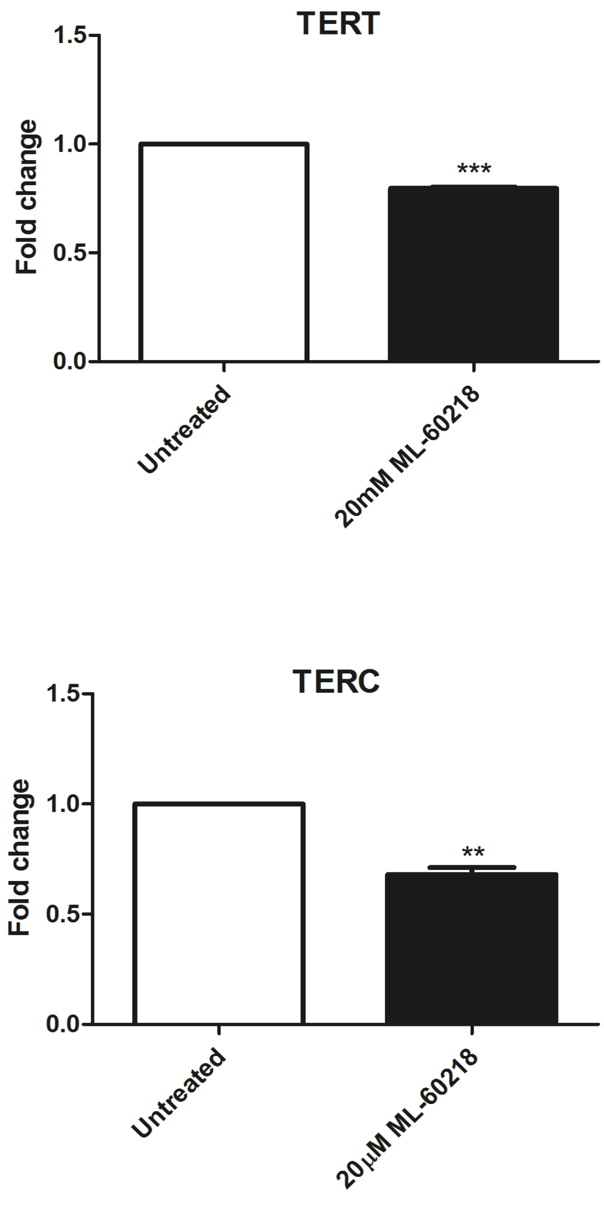

Supplementary Fig. 4

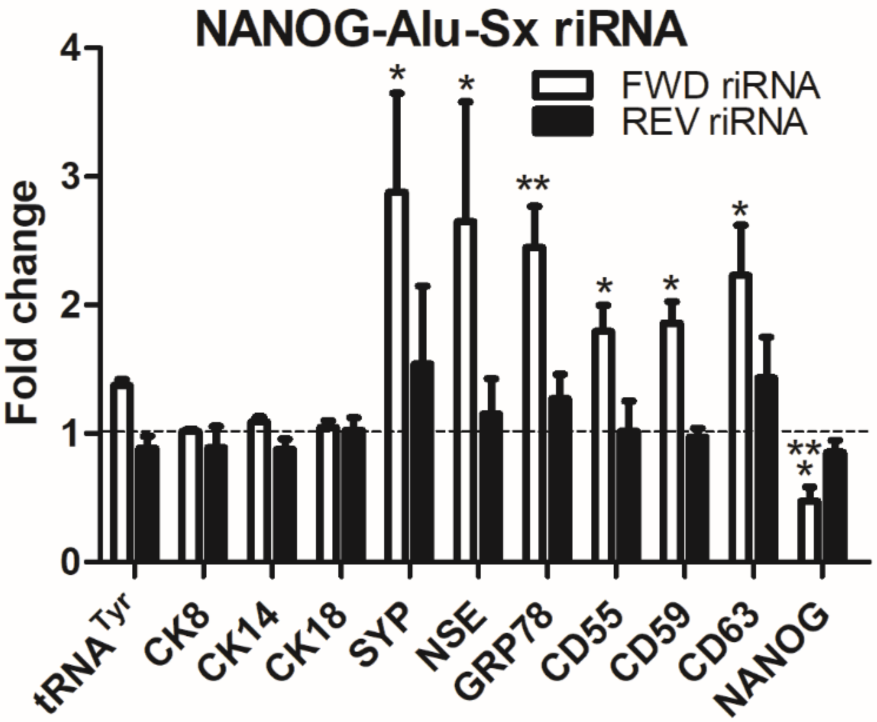

Supplementary Fig. 5

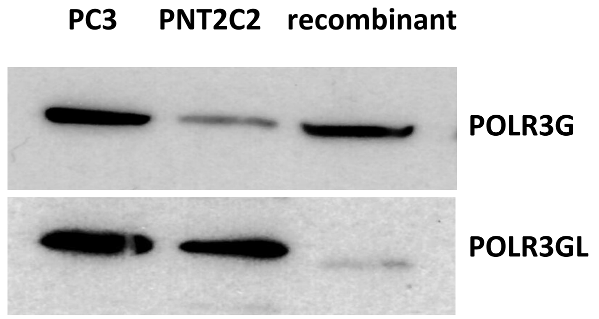

Supplementary Fig. 6

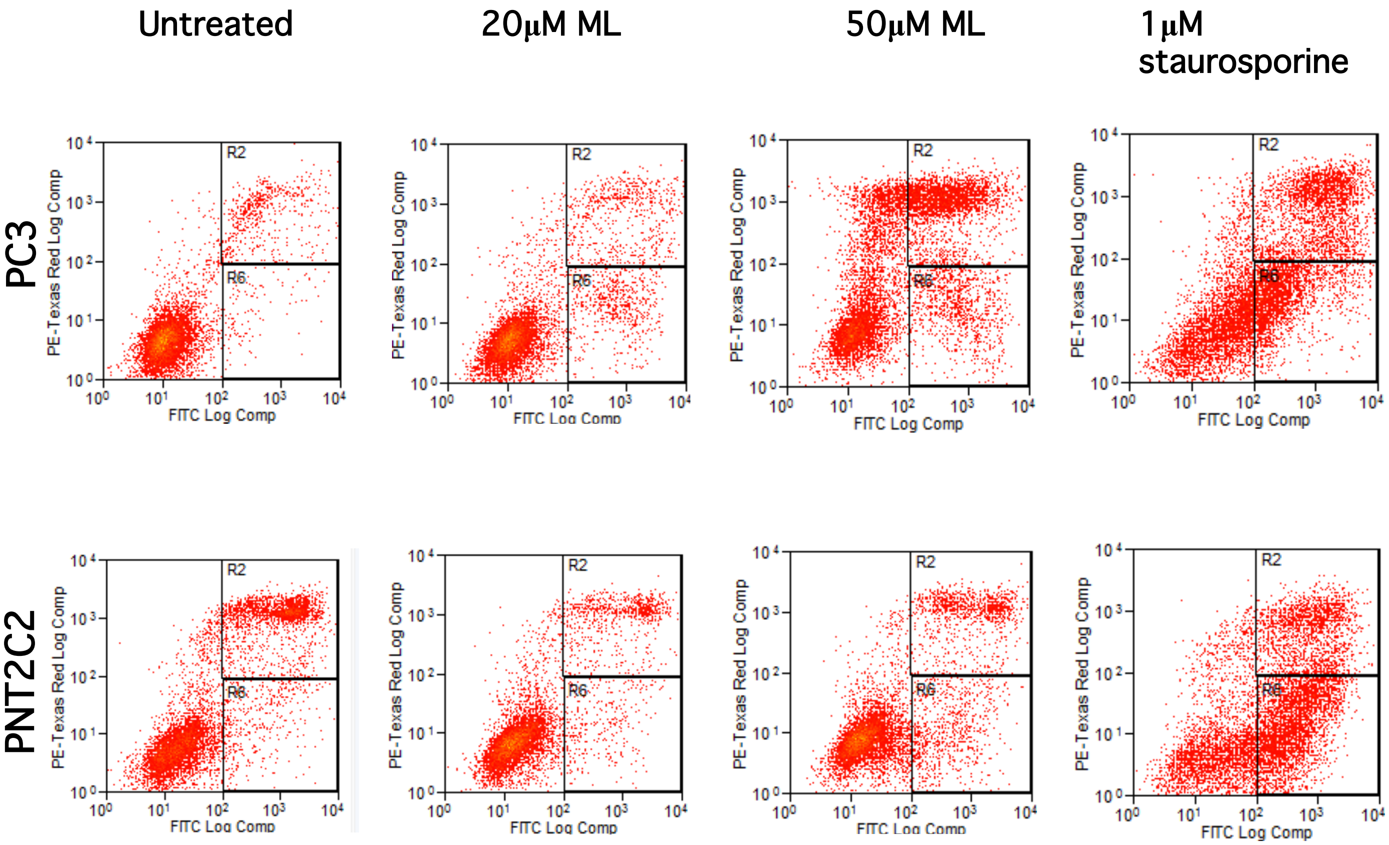

Supplementary Fig. 7

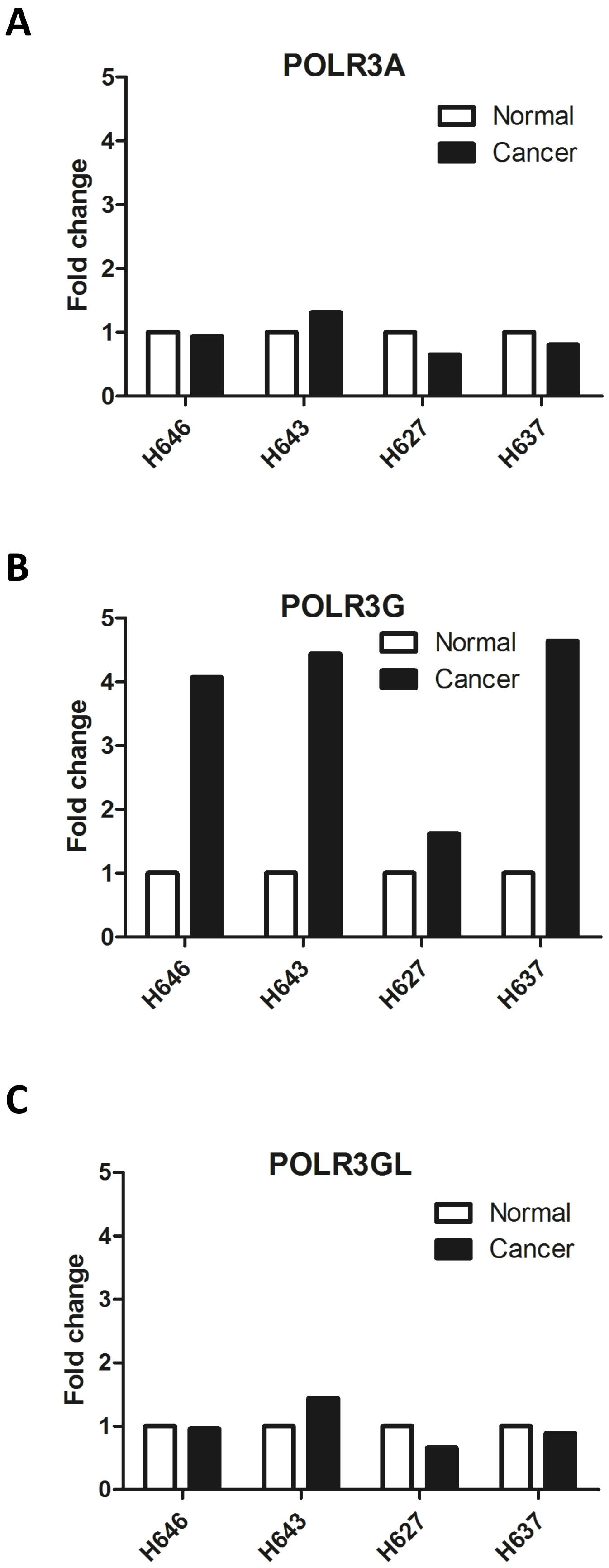

Supplementary Fig. 8

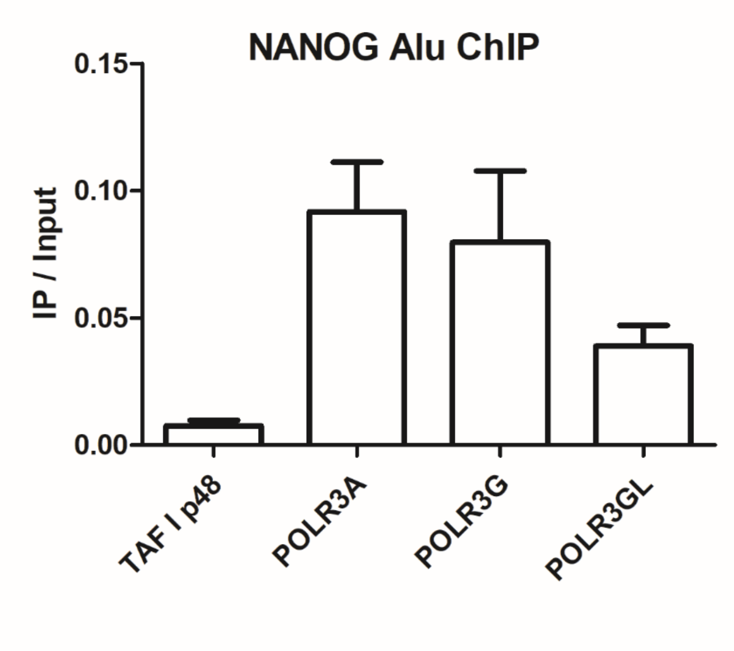

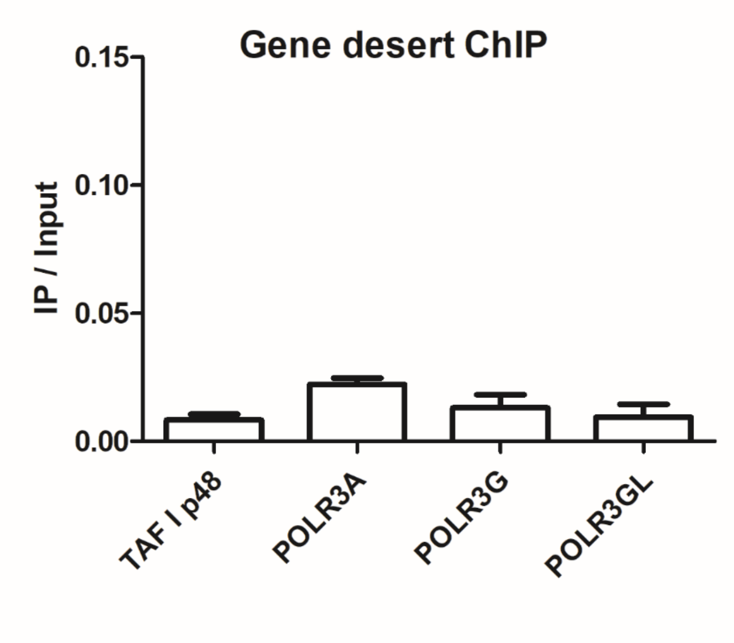

Supplementary Fig. 9

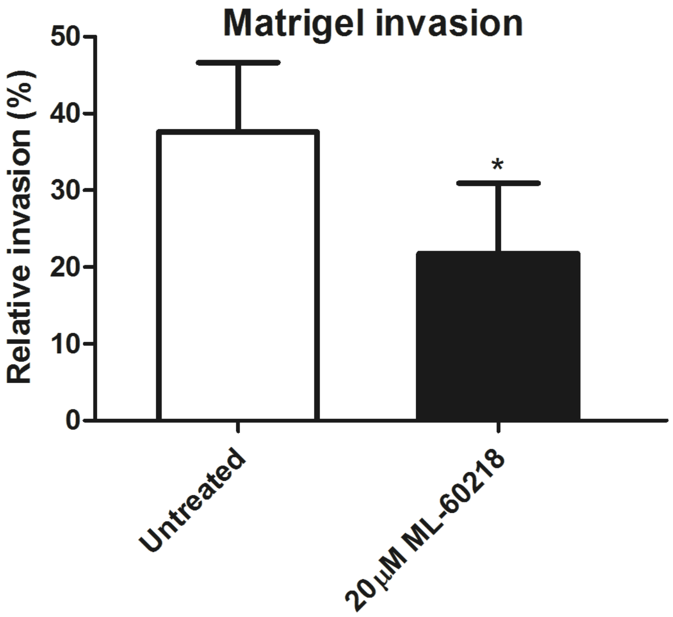

Supplementary Fig. 10

Pre-tRNA^{Arg}-CCG

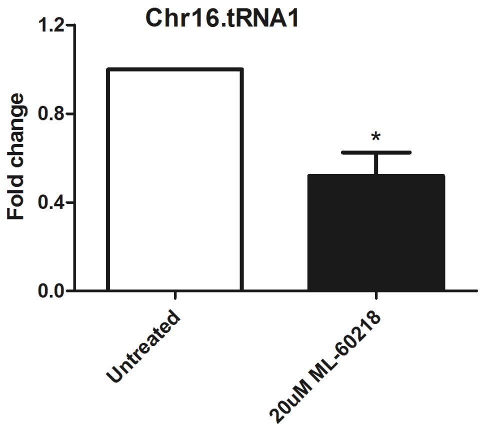

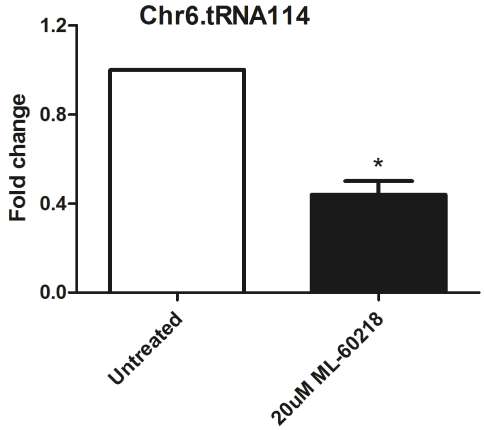

Pre-tRNA^{Glu}-UUC

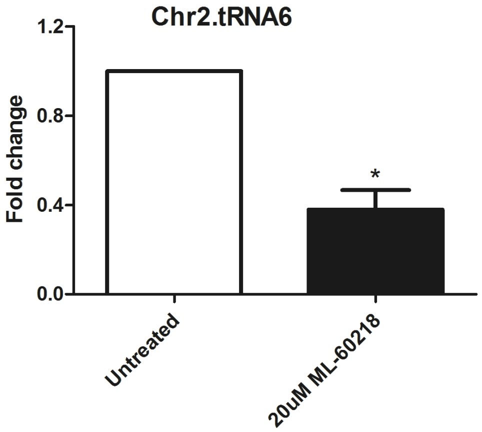

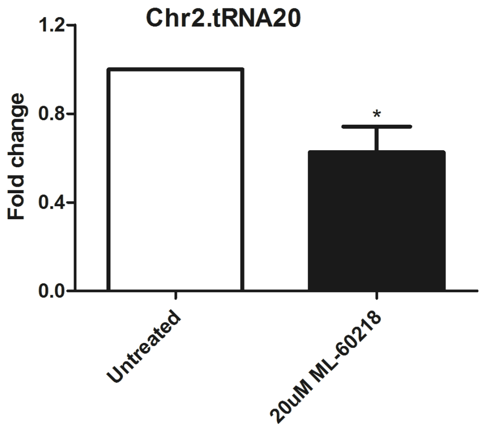

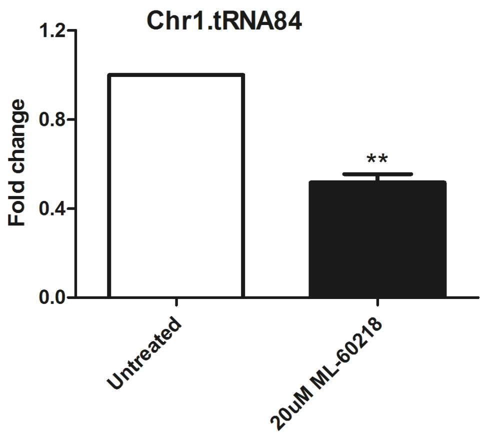

Supplementary Fig. 11

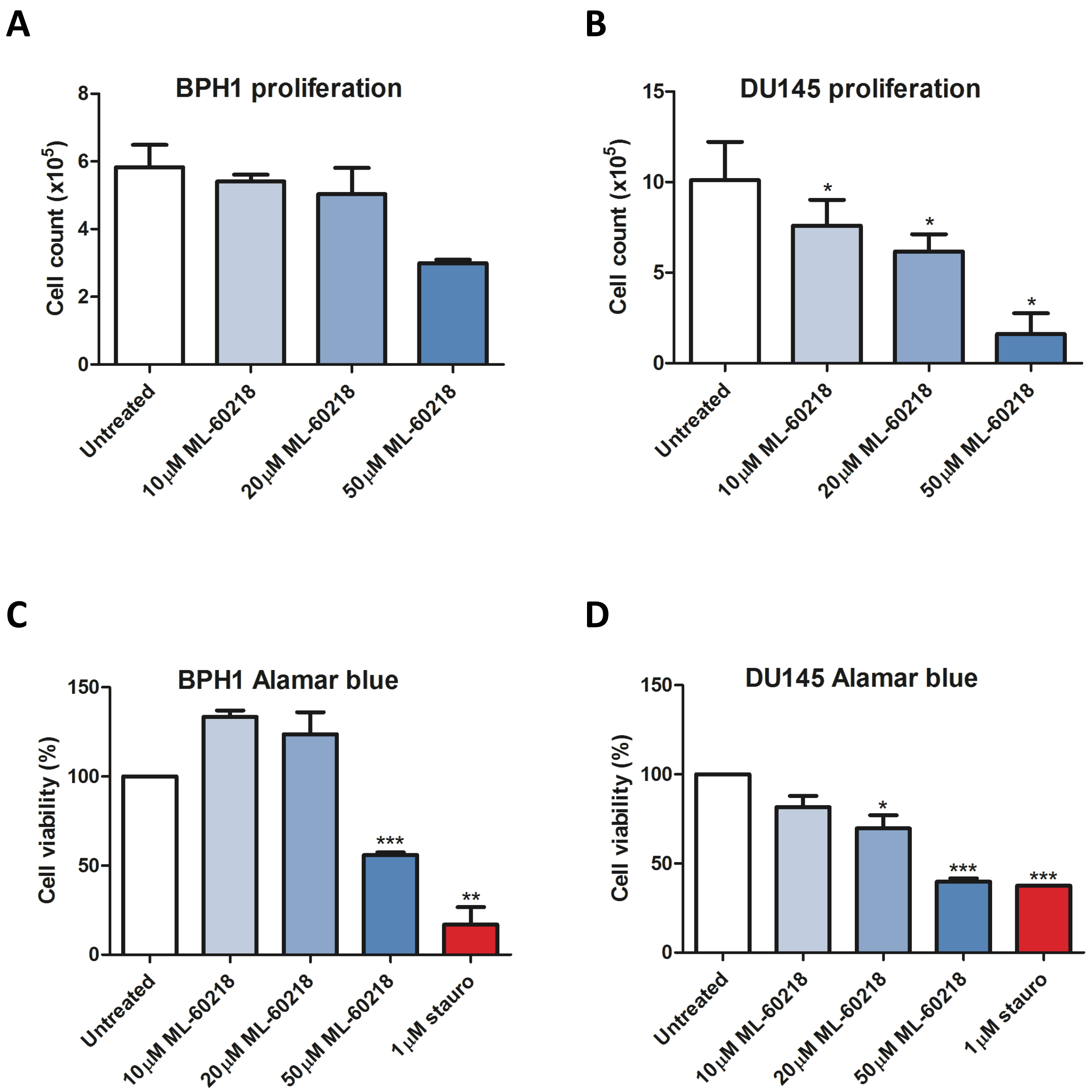

Supplementary Fig. 12

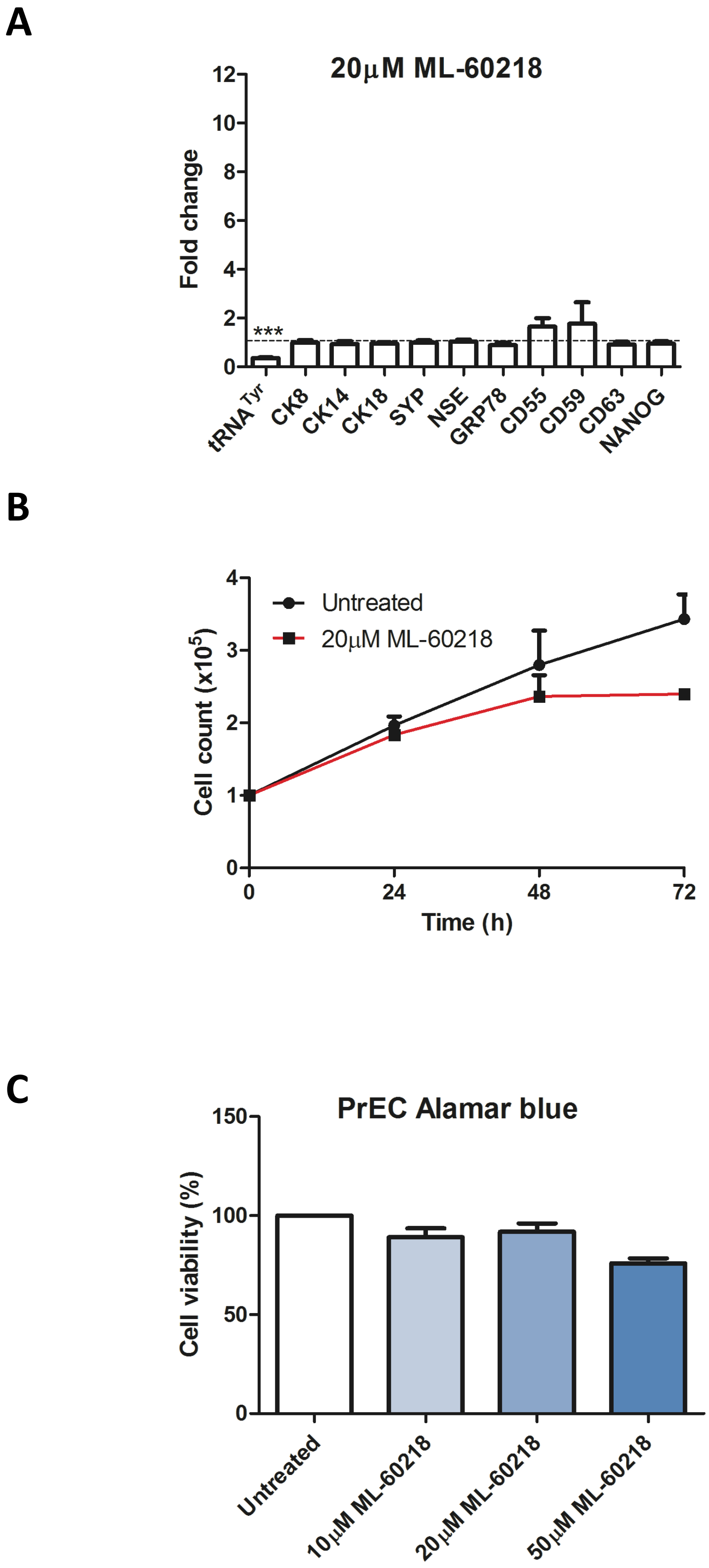

Supplement: Supplementary Data [file gkz128_supplemental_files.zip › Suppl Figs. Petrie et al. Revised .pdf]
